# Click Step-Growth Polymerization and *E*/*Z* Stereochemistry Using Nucleophilic Thiol–yne/–ene Reactions: Applying Old Concepts for Practical Sustainable (Bio)Materials

**DOI:** 10.1021/acs.accounts.2c00293

**Published:** 2022-08-25

**Authors:** Joshua C. Worch, Andrew P. Dove

**Affiliations:** School of Chemistry, The University of Birmingham, Edgbaston, Birmingham B15 2TT, U.K.

## Abstract

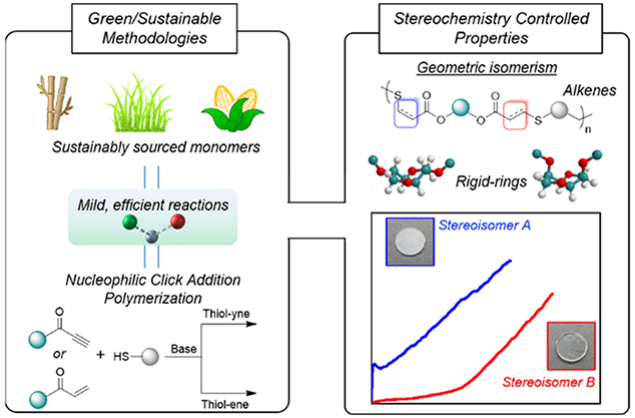

Polymer sustainability is synonymous with “bioderived polymers” and the zeitgeist of “using renewable feedstocks”. However, this sentiment does not adequately encompass the requirements of sustainability in polymers. In addition to recycling considerations and mechanical performance, following green chemistry principles also needs to be maximized to improve the sustainability of polymer synthesis. The synthetic cost (*i.e.*, maximizing atom economy, reducing chemical hazards, and lowering energy requirements) of producing polymers should be viewed as equally important to the monomer source (biomass vs petrol platform chemicals). Therefore, combining the use of renewable feedstocks with efficient syntheses and green chemistry principles is imperative to delivering truly sustainable polymers. The high efficiency, atom economy, and single reaction trajectories that define click chemistry reactions position them as ideal chemical approaches to synthesize polymers in a sustainable manner while simultaneously expanding the structural scope of accessible polymers from sustainably sourced chemicals.

Click step-growth polymerization using the thiol–yne Michael addition, a reaction first reported over a century ago, has emerged as an extremely mild and atom-efficient pathway to yield high-performance polymers with controllable *E*/*Z* stereochemistry along the polymer backbone. Building on studies of aromatic thiol–yne polymers, around 10 years ago our group began investigating the thiol–yne reaction for the stereocontrolled synthesis of alkene-containing aliphatic polyesters. Our early studies established a convenient path to high-molecular-weight (>100 kDa) *E-*rich or *Z*-rich step-growth polymers by judiciously changing the catalyst and/or reaction solvent. This method has since been adapted to synthesize fast-degrading polyesters, high-performance polyamides, and resilient hydrogel biomaterials. Across several systems, we have observed dramatic differences in material properties among polymers with different alkene stereochemistry.

We have also explored the analogous thiol–ene Michael reaction to create high-performance poly(ester-urethanes) with precise *E*/*Z* stereochemistry. In contrast to the stereoselective thiol–yne polymerization, here the use of monomers with predefined *E*/*Z* (geometric) isomerism (arising from either alkenes or the planar rigidity of ring units) affords polymers with total control over stereochemistry. This advancement has enabled the synthesis of tough, degradable materials that are derived from sustainable monomer feedstocks. Employing isomers of sugar-derived isohexides, bicyclic rigid-rings possessing geometric isomerism, led to degradable polymers with fundamentally opposing mechanical behavior (*i.e.*, plastic vs elastic) simply by adjusting the stereochemistry of the isohexide.

In this Account, we feature our investigation of thiol–yne/–ene click step-growth polymers and efforts to establish structure–property relationships toward degradable materials with practical mechanical performance in the context of sustainable polymers and/or biomaterials. We have paid attention to installing and controlling geometric isomerism by using these click reactions, an overarching objective of our work in this research area. The exquisite control of geometric isomerism that is possible within polymer backbones, as enabled by convenient click chemistry reactions, showcases a powerful approach to creating multipurpose degradable polymers.

## Key References

- BellC. A.; YuJ.; BarkerI. A.; TruongV. X.; CaoZ.; DobrinyinA. V.; BeckerM. L.; DoveA. P.Independent Control of Elastomer Properties through Stereocontrolled Synthesis. Angew. Chem., Int. Ed.2016, 55, 13076–1308010.1002/anie.201606750PMC508252027654023.^1^*This work established the stereocontrolled thiol–yne polymerization to afford high-molecular-weight (>100 kDa) E-rich or Z-rich step-growth polymers by varying the catalyst and/or reaction solvent. Stereochemical differences among polymers led to substantial changes to thermal and mechanical properties.*
- WorchJ. C.; WeemsA. C.; YuJ.; ArnoM. C.; WilksT. R.; HucksteppR. T. R.; O’ReillyR. K.; BeckerM. L.; DoveA. P.Elastomeric Polyamide Biomaterials with Stereochemically Tuneable Mechanical Properties and Shape Memory. Nat. Commun.2020, 11, 325010.1038/s41467-020-16945-832591525PMC7320000.^2^*This study extended the stereocontrolled thiol–yne polymerization to afford polyamides with advantageous properties. In contrast to conventional polyamides (nylons), the thiol–yne polyamides were amorphous and more processable but retained comparable mechanical strength. They also displayed high-fidelity shape-memory behavior.*
- MacdougallL. J.; Pérez-MadrigalM. M.; ShawJ. E.; WorchJ. C.; SammonC.; RichardsonS. M.; DoveA. P.Using Stereochemistry to Control Mechanical Properties in Thiol–Yne Click-Hydrogels. Angew. Chem. Int. Ed.2021, 60, 25856–2586410.1002/anie.202107161PMC929838934551190.^3^*In this study, we applied our stereocontrolled thiol–yne reaction to synthesize hydrogel biomaterials from propiolate- and thiol-functionalized multiarmed PEG precursors. The stereocontrolled hydrogels had similar physical parameters but different mechanical properties, which we used to study cell response and growth.*
- StubbsC. J.; WorchJ. C.; PrydderchH.; WangZ.; MathersR. T.; DobryninA. V.; BeckerM. L.; DoveA. P.Sugar-Based Polymers with Stereochemistry-dependent Degradability and Mechanical Properties. J. Am. Chem. Soc.2022, 144, 1243–125010.1021/jacs.1c1027835029980PMC8796236.^4^*This work describes the synthesis of high-performance poly(ester-urethane)s from stereopure isohexides using thiol–ene polymerization. Isomeric polymers had opposing mechanical behavior, i.e., plastic vs elastic. Copolymers and physical blends of isomeric polymers showed an unexpected relationship between degradability and mechanical properties.*

## 1 Introduction

Typically, polymers are synthesized through either step-growth or chain-growth mechanisms, both of which present limitations in the context of creating more sustainable plastics. While step-growth polymerization readily enables the introduction of heteroatoms into polymer backbones, which is important for recycling and performance considerations, the requirement for high-temperature (*>*200 °C) polycondensation for most leading polymers in this class means that the process is energy-intensive. On the other hand, most commercial chain-growth polymerizations typically occur at lower temperatures and are thus more energy-efficient. However, it is challenging to incorporate heteroatoms into the polymer backbones, without compromising the molar mass, mechanical properties, or uniform degradability of the materials. Recent advances in step-growth polymerizations have focused on replacing condensation reactions with low-temperature click chemistries.^5^ These reactions can offer advantages such as high efficiency to yield high-molar-mass polymers, orthogonality to enable the facile synthesis of functional materials, and, in some cases, rapid ambient temperature reactivity that lowers the energy demands of the process. Modern click step-growth polymerizations, including 1,3-dipolar cycloadditions,^6−9^ thiol/amino-epoxy reactions,^10,11^ sulfur(VI) fluoride exchange (SuFEx),^12,13^ nucleophilic aromatic substitution (S_N_Ar),^14,15^ and oxime formation^16^ demonstrate an array of methodologies by which to efficiently build polymers under mild conditions.

**Figure 1:**
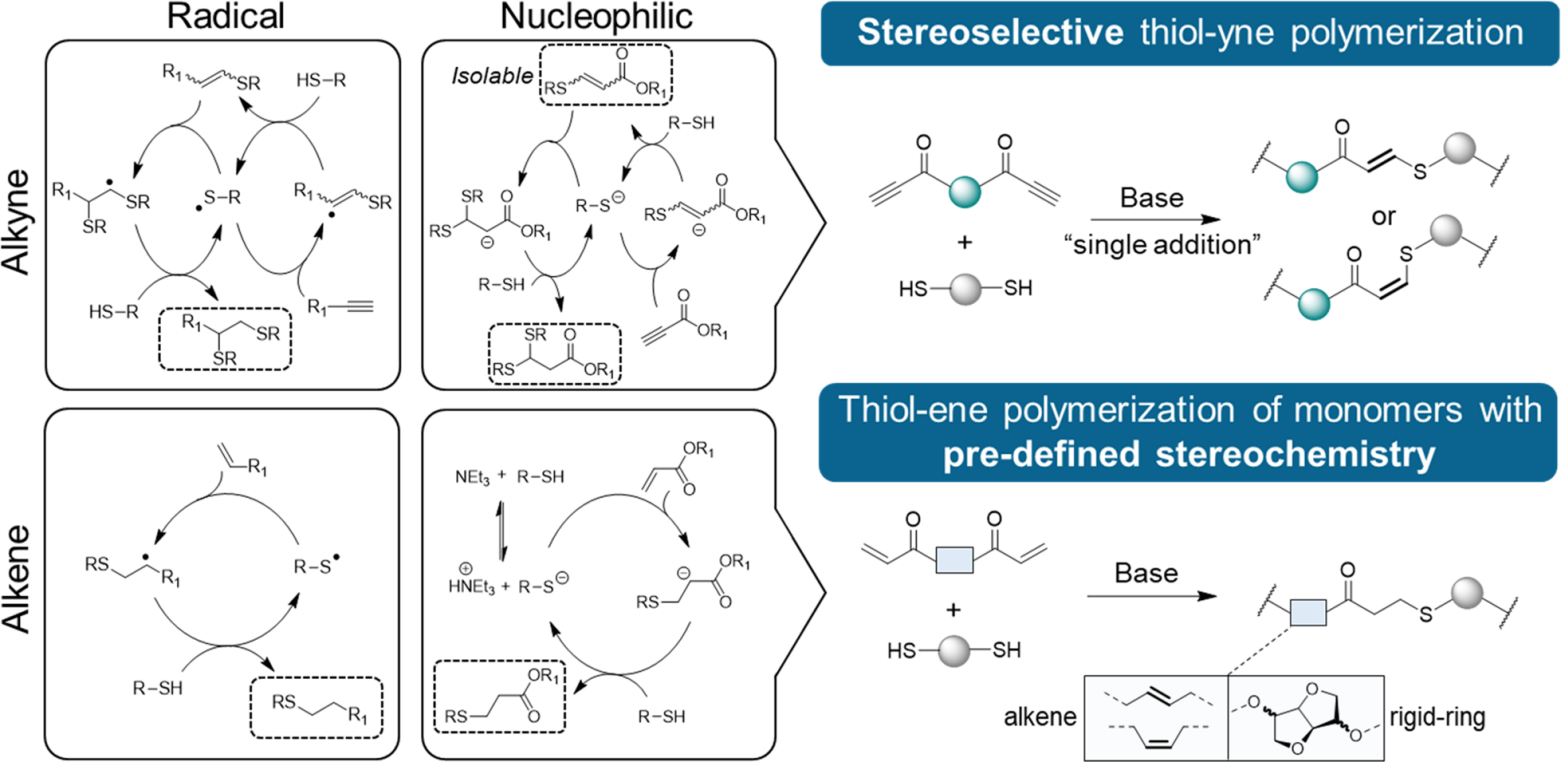
Nucleophilic and radical mechanisms for thiol addition to alkenes and alkynes (left) and nucleophilic thiol–yne/–ene polymerization to afford step-growth polymers with well-defined stereochemistry (right).

When our group became interested in using click chemistry to build polymers in the late 2000s, we were intrigued by conjugate addition reactions of nucleophiles to unsaturated moieties. At the time, the wider field was dominated by use of the radical-mediated thiol–ene reaction,^17,18^ which remains a powerful state-of-the-art technique in polymer synthesis and functionalization (Figure 1). Although the application of the analogous radical thiol–yne reaction^18−22^ in polymers was also developing, the radical thiol addition can lack regio- and/or stereocontrol^23,24^ and lead to unwanted side products.^24^ Moreover, two consecutive thiol additions across the alkyne may also occur to yield a thioacetal product, which was particularly advantageous for forming dendritic^19−22^ or cross-linked^12^ materials (Figure 1). While we had explored the base-catalyzed conjugate addition of thiols across activated double bonds (adjacent to an electron-withdrawing group) for polymer synthesis and end-group or side-chain functionalization,^25−29^ the analogous strategy using electron-deficient triple bonds was less studied (Figure 1).

However, the base-catalyzed thiol–yne Michael addition,^30^ first reported more than 100 years prior, was key to enabling us to adapt thiol–yne chemistry to achieve our goals by favoring a regio- and stereocontrolled single addition to a triple bond that would yield an unsaturated product. Specifically, we anticipated a versatile method to create structurally diverse heteroatom-rich polymers with controllable *E*/*Z* isomerism (Figure 1).

**Scheme 1:**
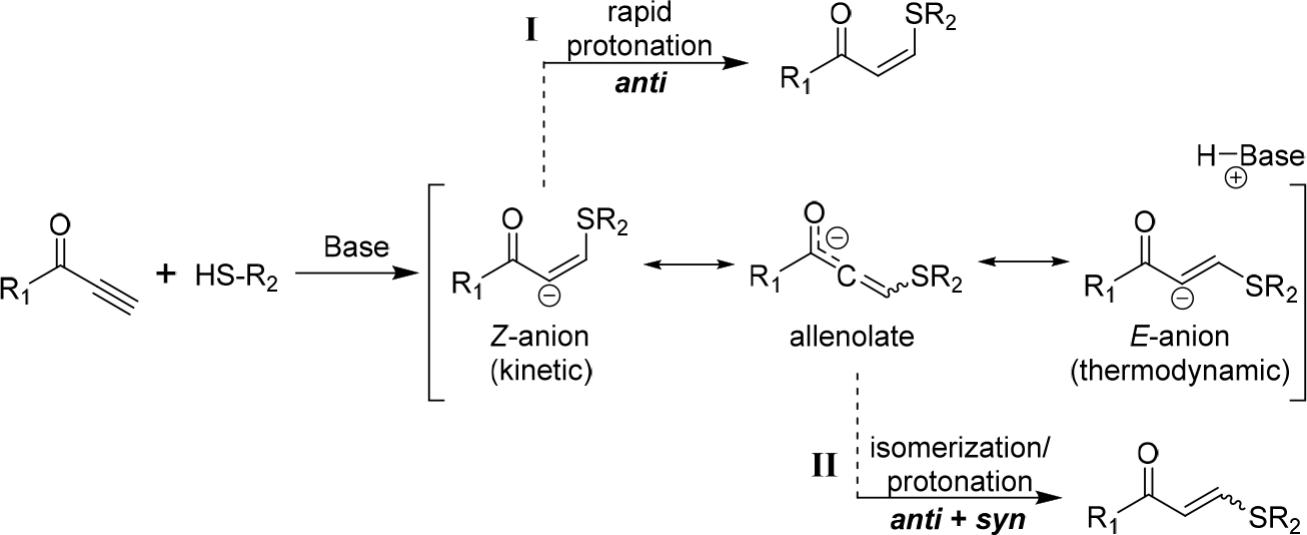
Proposed Mechanism for the Base-Catalyzed Thiol–yne Addition to Activated Acetylenes

The *E*/Z stereochemistry of thiol–yne Michael addition products was not investigated until the 1950s by Truce and co-workers.^31−33^ When employing thiolates in protic media, the *anti*-addition rule predominated to yield *Z*-isomer products. A more decisive study by the same group in the 1970s investigated the effect of electron-withdrawing groups (ester, amide, nitrile, *etc.*).^34^ Again, for most of the substrates the thiolate addition in protic media proceeded *via* nucleophilic attack at the β-carbon to generate a *Z*-anion followed by rapid protonation of the α-carbon to yield 100% *Z*-products (pathway I, Scheme 1). However, reactions employing substrates with carbonyl-containing activating groups, such as propiolates, violated this rule to yield products with mixed stereochemistry (resulting from both *syn*- and *anti*-additions) (pathway II, Scheme 1). The authors speculated that this was due to the formation of an allenolate intermediate which could facilitate isomerization. Although this study reported quantitative yields when employing stoichiometric thiolates, procedures that featured catalytic amounts of base (such as triethylamine, NEt_3_) were not as efficient.

Subsequent work in the following decades generally supported these historic findings where the *anti*-addition rule favored *Z*-products, especially in protic or highly polar solvents.^35−39^ Similarly, these studies also generally showed that acetylenes featuring electron-withdrawing groups that could stabilize the negative charge of the anionic intermediates were less *Z-*selective. In some cases, the use of these highly activated acetylenes even led to quantitative conversion at ambient temperature when using only a catalytic amount of base.^35,37,39^ Solvent effects on stereoselectivity were also described in these studies with the proton-donating ability of the solvent being important. Protic solvents can rapidly protonate the kinetic *Z*-anion (Scheme 1), while equilibration of the allenolate intermediate to the more thermodynamically stable product (greater *E*-selectivity) may occur when using nonpolar aprotic solvents. These principles would serve to influence our initial work in adapting the reaction to polymer synthesis.

Our work in utilizing the base-catalyzed nucleophilic thiol–yne reaction for the stereocontrolled synthesis of unsaturated polymers began in 2013 with our report on the chain–chain coupling between propiolate- and thiol-terminated small molecules and PEG oligomers.^40^ In that work, we established the high efficiency and stereoselectivity of the reaction. We found that catalytic amounts of a commercial amine (such as NEt_3_) or amidine (such as 1,8-diazabicyclo[5.4.0]undec-7-ene (DBU)) led to the quantitative formation of α,β-unsaturated products with opposing stereochemistry. When reactions were conducted in chloroform, the use of afforded *E*-isomer products (up to 97%) and the analogous reactions with DBU led to high *Z*-content (up to 80%). This result was striking and not entirely explained by historic mechanistic interpretations. (See Scheme 1 and the accompanying discussion.) A cohesive mechanistic description for the catalyst effect on stereoselectivity remains unknown. We also found that the solvent choice was also critical to the stereochemistry and kinetics of the thiol–yne addition, in accordance with previous reports.^37^ High dielectric solvents increased the reaction rate and promoted *Z*-isomer products, but less-polar solvents were more sluggish, yet still efficient (<1 h of reaction time), and favored *E*-isomer product formation. Concurrent optimization of both solvent and catalyst allowed for the targeted synthesis of both *E*- and *Z*-isomers with very high selectivity (>98%).

By applying these observations, we anticipated a path to high-molecular-weight unsaturated polyesters with controllable stereochemistry. Most importantly, the operational simplicity of the reaction was apparent. The base-catalyzed thiol–yne additions are tolerant of wet solvents and oxygen, which allows for benchtop reactions in the open atmosphere. Together, the accessibility that these features makes the practical application of the thiol–yne reaction highly attractive to a range of stakeholders.

## 2 Stereocontrolled Thiol–yne Reactions for Step-Growth Polymers

Stereochemistry is an essential aspect of molecular function and is equally impactful to the properties of polymers.^41^ The control of chirality, or tacticity, of pendant groups in polymer chains has been used to great effect to manipulate and enhance the thermomechanical performance of commercially important modern plastics, with notable examples including isotactic polypropylene and poly(lactic acid).^41^ Conversely, installing and/or controlling *E*/*Z* isomerism in synthetic polymers is comparatively underdeveloped and, as such, is not frequently encountered in commodity materials. In fact, it remains largely uncharted despite the remarkable differences that are observed between isomers of vulcanized natural polyisoprene: the *Z*-isomer (natural rubber) is elastic and soft, yet the *E*-isomer (gutta-percha) is brittle and hard.^42,43^ However, synthetically mimicking the stereocontrol found in natural polyisoprenes is challenging and even more so when attempting to access polymers with intermediate stereochemistry and properties. The most well-known examples include the synthesis of stereoblock polyisoprenes^44,45^ among other unsaturated polyolefin structures^46^ which can imbue the resultant polymers with thermoplastic elastomeric behavior.

The distinctive behavior between isomeric polyisoprenes inspired our group’s interest in *E*/*Z*-alkene isomerism in polymers. In the early 2010s, the majority of the stereocontrolled unsaturated synthetic polymers possessed all-carbon backbones (***e.g.***, polybutadiene, polyneoprene, and polynorbornene) and were usually synthesized *via* metal-catalyzed chain-growth mechanisms, although some unsaturated step-growth polymers synthesized from inefficient polycondensation reactions were also known.^41^ Thus, we viewed the manipulation of double-bond stereochemistry in unsaturated polymers as an underdeveloped area. Specifically, there was an opportunity to develop alkene-containing polar polymers (*i.e.*, polyesters) using click step-growth polymerizations. This would provide a strategic advantage over inefficient polycondensations that necessitate harsh reaction conditions, which typically result in the isomerization of double bonds, thus limiting the stereochemical control within unsaturated polymers.

Notably, work in the late 1980s reported several base-catalyzed thiol–yne polymerizations using thiophenols and aromatic ynones, although their poor solubility generally precluded in-depth characterization.^47−49^ In 2010, Tang and co-workers also reported an amine-catalyzed thiol–yne polymerization of bisthiophenols and aromatic dipropiolates to yield unsaturated step-growth polymers with moderate molar mass (*M*_W_ = 30 kDa) and high *Z*-content (up to 80%).^50^ While these studies had an exclusive focus on aromatic polymers, we anticipated that the aliphatic polymers would have superior processability, mechanical performance, and degradability, all of which are important metrics to consider in the context of sustainable polymers. A rhodium-catalyzed stereoselective thiol–yne polymerization to afford aromatic polymers was reported in 2011;^51^ however, a full investigation of stereochemistry–property effects was not conducted. Instead, we sought to employ organocatalysts for the same purpose and fully exploit the stereocontrol of the reaction to modulate polymer thermomechanical properties.

**Figure 2:**
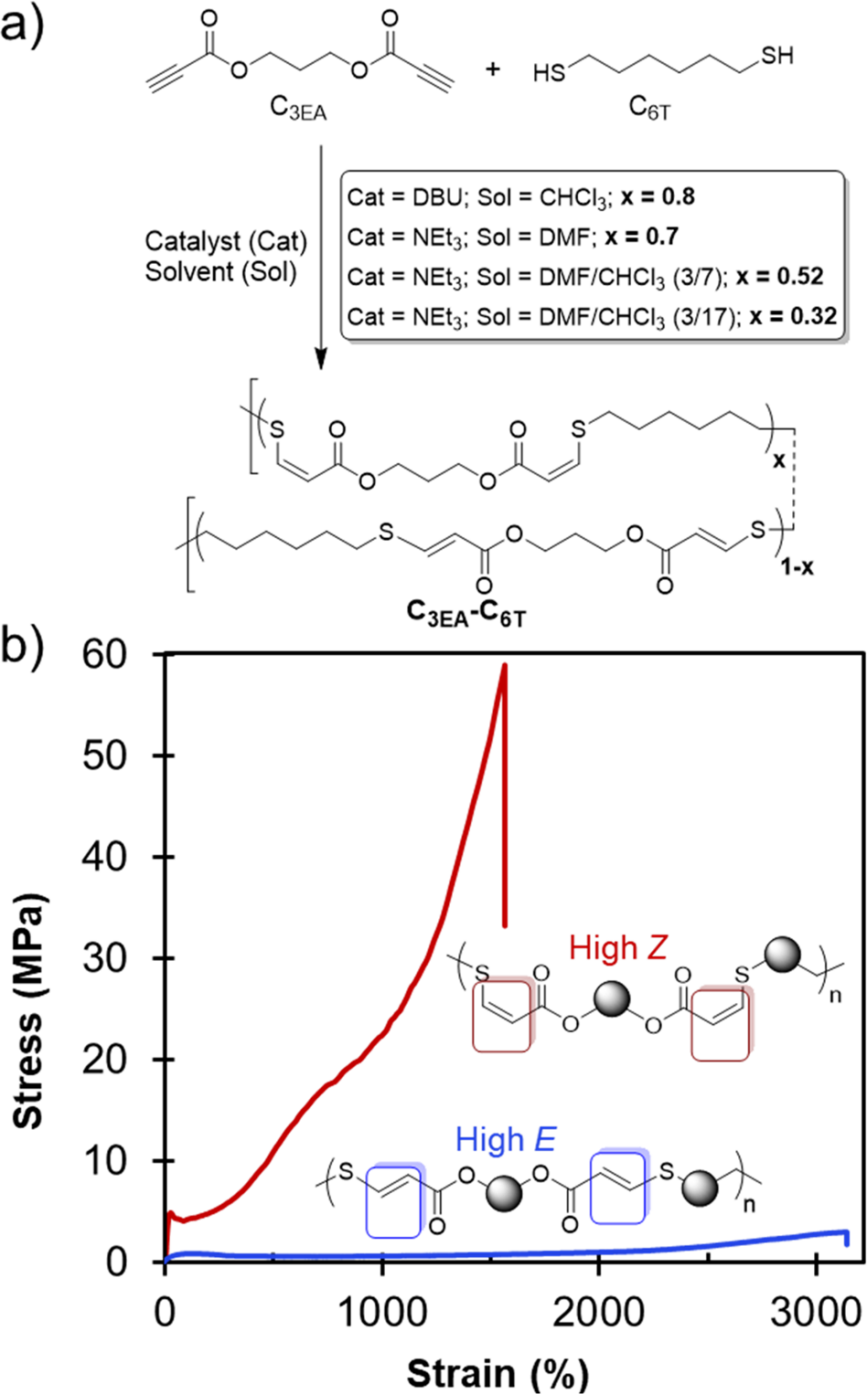
(a) Synthesis of thiol–yne materials from dialkyne and dithiol precursors. (b) Exemplar stress vs strain data for polymers with high *Z*- and high *E*-alkene content. Polymer data: high *Z* (80% *Z*), *M*_w_ = 148 kDa and *Đ*_M_ = 5.6; high *E* (32% *Z*), *M*_w_ = 125 kDa and *Đ*_M_ = 3.68. Adapted with permission from ref (1). Copyright 2016 The Authors. Published by Wiley under the Creative Commons Attribution 4.0 International (CC BY 4.0) License.

In 2016, we published a study on the synthesis of stereocontrolled thiol–yne polyesters from aliphatic dipropiolates and commercially available dithiols.^1^ Polymers with high molar mass (*M*_W_ > 100 kDa) were synthesized at ambient temperature in just 1 h using 1 mol % catalyst loading (NEt_3_ or DBU) which corroborated the click-like nature of the reaction. Studying a system synthesized from propane-1,3-diyl dipropiolate (C_3EA_) and 1,6-hexanedithiol (C_6T_) yielded polymers with interesting mechanical properties, and initial studies into the effect of *E*/*Z* stereochemistry on mechanical properties were further investigated using this composition. When various catalyst/solvent combinations (chloroform and/or dimethylformamide) were used, the stereochemistry was changed from 32 to 80% *Z*-content. Although all of the C_3EA_–C_6T_ polyesters could be broadly characterized as thermoplastic elastomers with distinct yield points, there were stark stereochemical differences among their behavior (Figure 2).

Polymers with *Z*-content ≥70% were semicrystalline and extremely tough (1500% elongation at break and ultimate tensile strength near 60 MPa for 80% *Z* polymers). On the other hand, polymers with 32 and 53% *Z*-content were amorphous and more extensible, but at the cost of decreased tensile strength and Young’s modulus (an order of magnitude lower). These mechanical behaviors remained consistent even when the polymer molecular weight and/or dispersity (*Đ*_M_) varied. The stereochemistry–property relationship (*E* is weaker and amorphous, *Z* is tougher and semicrystalline) was surprising and at odds with polyisoprene data, where *E*-isomer polymers are stronger and more crystalline. Infrequently, we have reached 15–90% *Z* content in related polymers, but we have not observed significant differences in the thermomechanical–property relationships previously described for materials containing 30–80% *Z* isomers.

**Figure 3:**
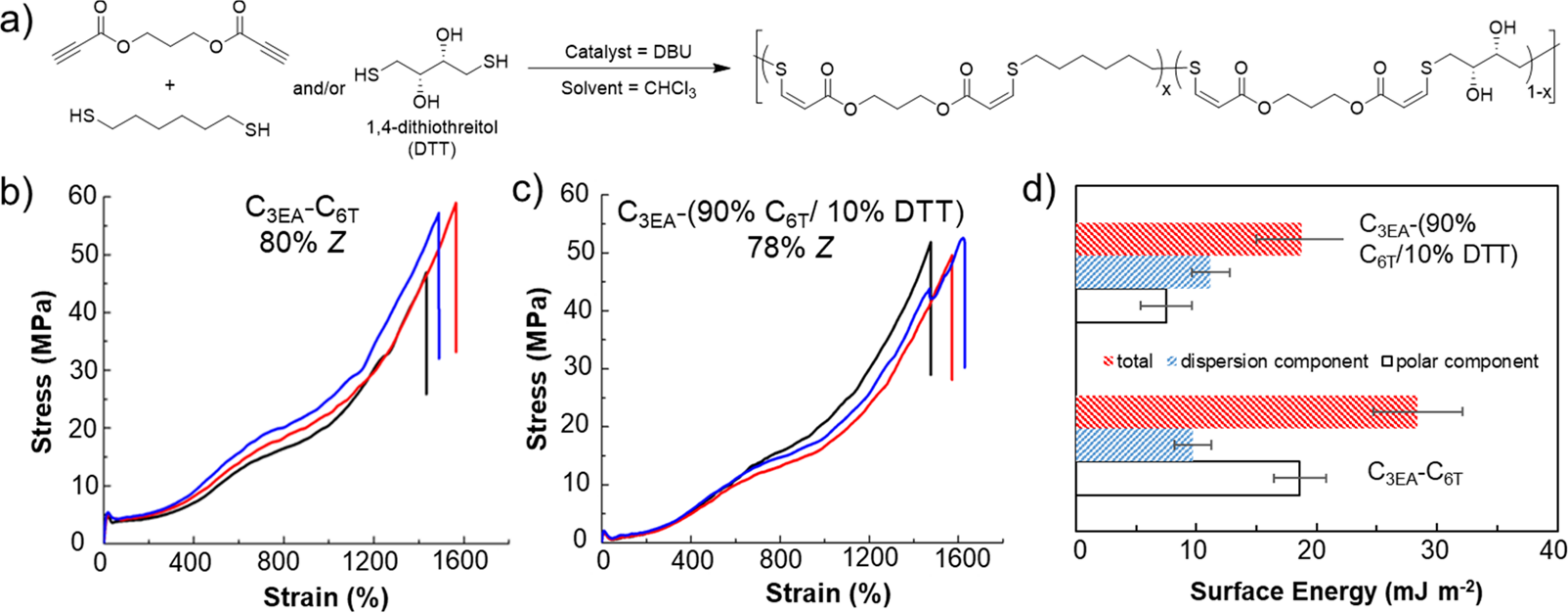
(a) Synthesis of thiol–yne copolymers from dialkyne and dithiol precursors. (b) Exemplar stress vs strain curves for the thiol–yne polymer with 80% *Z*-alkene content composed of propane-1,3-dipropiloate (C_3EA_) and 1,6-hexanedithiol (C_6T_). (c) Exemplar stress vs strain curves for thiol–yne polymer with 78% *Z*-alkene content composed of C_3EA_ and 90% C_6T_/10% DTT. Data for three samples are shown to illustrate the reproducibility. (d) Surface energy data for C_3EA_–C_6T_ and C_3EA_–(90% C_6T_/10% DTT). Polymer data: C_3EA_–C_6T_ (80% *Z*) *M*_w_ = 148 kDa and *Đ*_M_ = 5.6; (78% *Z*) *M*_w_ = 110 kDa and *Đ*_M_ = 3.77. This figure was produced using data taken from ref (1).

**Figure 4:**
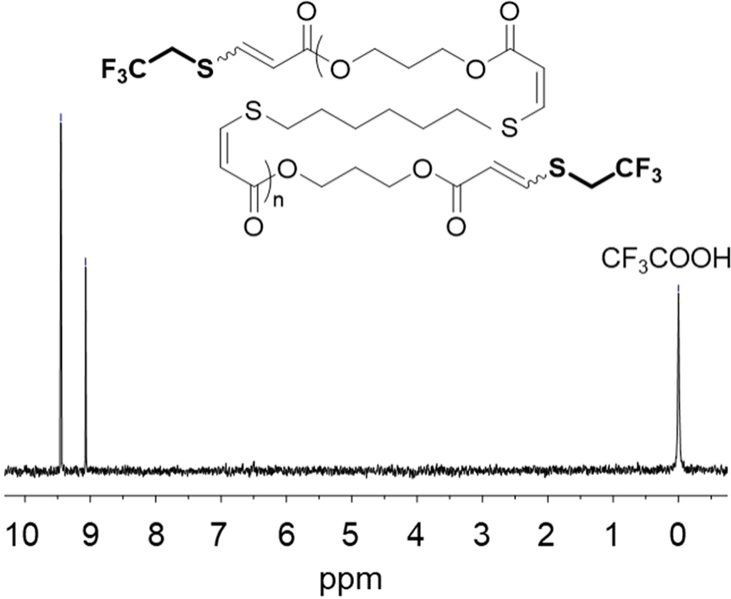
^19^F NMR spectrum of the C_3EA_–C_6T_ thiol–yne step-growth polymer following end-capping with 2,2,2-trifluoroethanethiol (376 MHz, CDCl_3_ + 0.01% v/v CF_3_COOH). Adapted with permission from ref (1). Copyright 2016 The Authors. Published by Wiley under the Creative Commons Attribution 4.0 International (CC BY 4.0) License.

The highly selective reaction trajectory of the nucleophilic thiol–yne addition also enabled us to control other material properties through judicious monomer design and end-group modification. The surface properties of the materials can be modified independently from the bulk properties by introducing 1,4-dithiothreitol (DTT) as a comonomer (Figure 3). Even at loadings of as low as 10% of the thiol comonomer content, the polar component of the surface energy was observed to be significantly reduced, while the mechanical properties did not change significantly. Attempts to modify the double bond by either subsequent thiol addition or other chemical reactions have largely led to rapid polymer degradation. Despite this, cross-linking with 1 wt % dicumyl peroxide at 160 °C in the bulk led to materials that displayed a reduced ultimate tensile strength but greater elastic recovery. Typically, we use a slight excess of dipropiolate monomer in these polymerizations to control the molar mass of the final polymer through stoichiometric imbalance (following the modified Carother equation). We believe that this approach provides the most stable end-group as there is a risk of disulfide formation or further reaction of thiol end-groups. In turn, this presents the opportunity to selectively functionalize the end-groups of these high-molar-mass polymers through a targeted addition, demonstrated with 2,2,2-trifluoroethanethiol (Figure 4).

Further attempts to create materials with additional functional groups focused on the incorporation of an internal unactivated alkyne between the propiolate reactive groups.^52,53^ As anticipated, this did not interfere with the polymerization process but instead provided a handle that could be either selectively functionalized or cross-linked through Ru-catalyzed alkyne–azide cycloaddition chemistry. Finally, in order to create a noncovalent network through hydrogen bonding in the materials, a dipropiolate monomer that contained urethane linkages was created and polymerized.^54^ While stereochemical effects on the mechanical performance were still clear, the hydrogen bonding led to strong elastomers at high *Z*-contents and the materials possessed thermally stimulated shape memory behavior (recovery of their original shape after deformation).

Our attempts to directly functionalize the backbone alkenes of various thiol–yne polyesters (**e.g.**, C_3EA_–C_6T_) using radical and/or nucleophilic additions have been largely unsuccessful. We typically observed a large decrease in the molecular weight of the polymer, indicative of backbone degradation. Despite these observations, the thiol–yne polyesters were found to be highly resistant to hydrolysis under basic conditions. Indeed, unpublished data from our laboratory indicates that these polymers have exceptional hydrolytic stability (<1% mass loss after 1 year in strongly alkaline solution).

**Figure 5:**
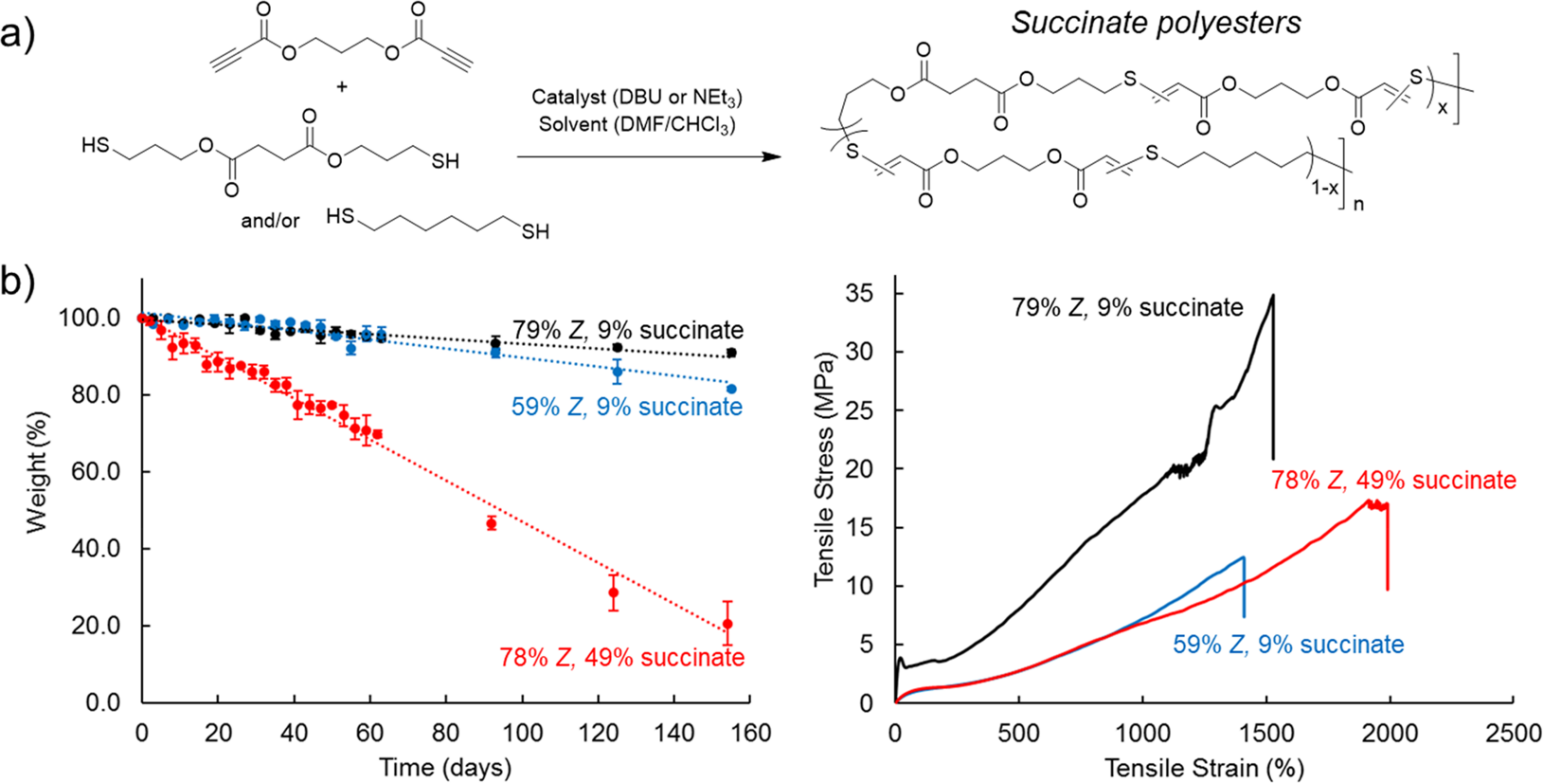
(a) Synthesis of succinate-containing thiol–yne materials from dialkyne and dithiol precursors. (b) Demonstration of independent control over degradability (weight loss vs time for samples under accelerated hydrolysis conditions) and mechanical properties (stress vs strain curves) for polymers with different succinate monomer content and/or alkene stereochemistry. Polymer data: 79% *Z* (9% succinate), *M*_w_ = 111 kDa and *Đ*_M_ = 3.74; 59% *Z* (9% succinate), *M*_w_ = 117 kDa and *Đ*_M_ = 3.42; 59% *Z* (9% succinate), *M*_w_ = 124 kDa and *Đ*_M_ = 2.36. Adapted with permission from ref (52). Copyright 2021 The Authors. Published by Springer Nature under the Creative Commons Attribution 4.0 International (CC BY 4.0) License.

We became interested in increasing the hydrolytic degradability of the thiol–yne polyesters to further the materials from both sustainable polymers and biomaterials angles. This led us to further explore copolymer formulations that incorporated an aliphatic ester which would be expected to increase the hydrolytic degradability of the polymers (Figure 5).^52^ There are numerous commercial dithiols yet most are simple aliphatic and hydrocarbon-based. Thus we synthesized hydrolytically labile dithiol by esterifying succinic acid with 3-mercaptopropanol. Due to the operational simplicity of the thiol–yne polymerization, polymers of C_3EA_–C_6T_ composition were systematically produced to include succinate units (0–100%) by altering the stoichiometry of the two distinct dithiol monomers. As anticipated, the degradation kinetics were positively correlated to succinate content (70% mass loss after 10 days in strong alkaline solution). The *in vitro* degradation proceeded *via* a surface erosion process to afford linear degradation profiles. This behavior was translated to *in vivo* studies to reveal a fully resorbable biomaterial with minimal inflammatory and cytotoxicity response markers. Simultaneously, the stereochemistry of the polymers could be controlled by adjusting the reaction conditions. Hence, we could independently tune the degradability and mechanical properties by combining compositional and stereochemical control (*i.e.*, one can produce two materials with similar mechanical strength but different degradation rates or *vice versa*). This addressed a longstanding challenge in polymer biomaterials (and degradable polymers, more generally speaking): the inability to decouple degradability from mechanical properties. Previously, this concept had been demonstrated only in hydrogels, which are swollen and highly cross-linked polymer networks.^55,56^ The importance of this feature lies in the fact that biomaterials need to be exquisitely tailored to the wide range of mechanical requirements found in biological tissues, and the biomaterial residence time *in vivo* needs to be equally harmonized.

**Figure 6:**
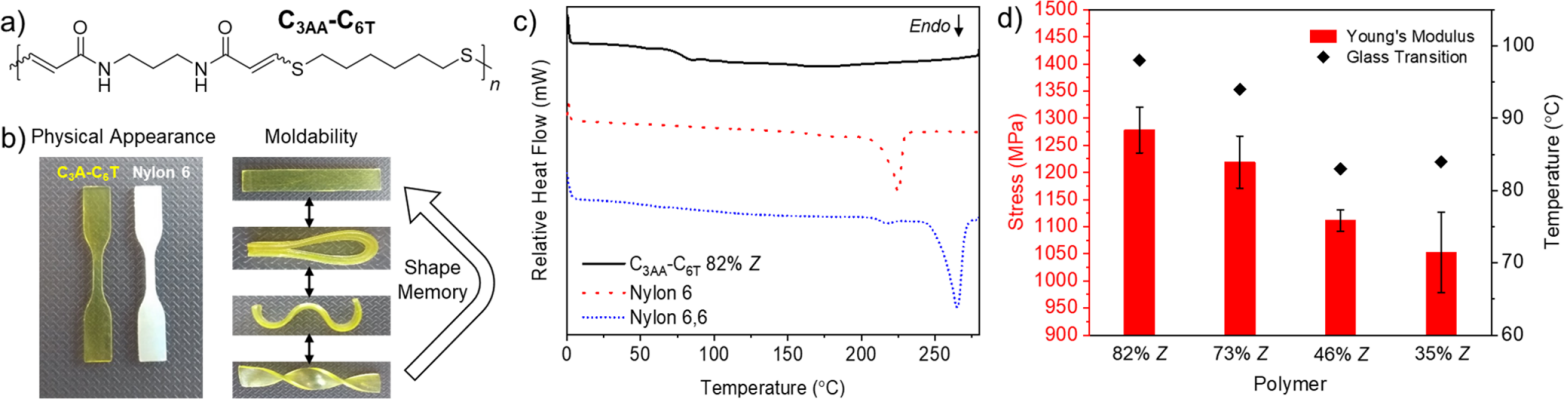
(a) Structure of polyamide C_3AA_–C_6T_. (b) Images to show the physical appearance and moldability of C_3AA_–C_6T_ (nylon 6 included for comparison). (c) DSC thermograms of the first heating cycle for C_3AA_–C_6T_, nylon 6, and nylon 6,6 to demonstrate the amorphous nature of C_3AA_–C_6T_. (d) Bar chart to show how Young’s modulus and the change for C_3AA_–C_6T_ at different *E*/*Z* ratios. Error bars represent 1 s.d. Polymer data for C_3AA_–C_6T_: 82% *Z*, *M*_w_ = 105 kDa and *Đ*_M_ = 3.35; 73% *Z*, *M*_w_ = 131 kDa and *Đ*_M_ = 3.39; 46% *Z*, *M*_w_ = 112 kDa and *Đ*_M_ = 3.84; 35% *Z*, *M*_w_ = 113 kDa and *Đ*_M_ = 4.7. Adapted with permission from ref (2). Copyright 2021 The Authors. Published by Springer Nature under the Creative Commons Attribution 4.0 International (CC BY 4.0) License.

Pivoting from polyesters, we explored the nucleophilic thiol–yne polymerization to synthesize high-molar-mass (>100 kDa) and high-*T*_g_ (up to approximately 100 °C) polyamide thermoplastics (Figure 6).^2^ We initially screened the DBU-catalyzed (1 mol %) reaction of an analogous three-carbon dipropiolamide (C_3AA_) with C_6T_ in dimethyl sulfoxide (DMSO) to afford 73% *Z* polyamides. The *Z*-selectivity could be increased to 82% when using DMSO/methanol solvent mixtures. However, obtaining high-*E* polyamides was more challenging since *E*-isomers are favored with weaker bases in nonpolar solvents for the thiol–yne reaction. NEt_3_ was found to be a poor catalyst for the propiolamide-thiol addition, and DMSO, which is a highly polar solvent, was found to be necessary to ensure good polymer solubility. However, by employing 1,4-diazabicyclo[2.2.2]octane (DABCO) at higher loadings (10 mol %) and adding chloroform as a co-solvent, we could isolate high-molar-mass polymers with 35% *Z*-selectivity. Interestingly, all of the melt-pressed thiol–yne polyamides were amorphous regardless of stereochemistry. This was unexpected considering the high degree of crystallinity that is typically observed in other synthetic polyamides such as nylons. Nevertheless, there was still a clear stereochemistry–property relationship. Generally, the high-*Z* polymers had a greater *T*_g_ (Δ = 14–15 °C) and Young’s modulus (∼20% higher), but at the cost of reduced ductility.

In this case, their amorphous nature was advantageous since it imbued the polyamides with a high degree of processability as compared to semicrystalline nylons that possess comparable mechanical profiles. Above all, the most interesting feature was their high-fidelity shape memory behavior and elasticity, which we serendipitously discovered when physically manipulating the polymer films into different shapes at various temperatures. Due to their excellent processability and physical adaptability coupled with their outstanding mechanical strength, we pursued the polyamides as nonresorbable (nonbiodegradable) biomaterials and found excellent long-term stability and compatibility *in vivo*.

## 3 Thiol–yne Hydrogel Materials

In the early 2010s, the use of click chemistry to make network polymers, including hydrogels, was expanding. At the time, the nucleophilic thiol–ene reaction using various Michael acceptors (including maleimide, vinyl sulfones, and acrylates) was already established as a mild and efficient method to synthesize hydrogel materials.^57^ However, the analogous nucleophilic thiol–yne reaction was yet to be investigated in this context. We envisioned that the thiol–yne reaction would be excellently suited for hydrogel synthesis as both functional groups were tolerant of water and due to the high efficiency of uncatalyzed reactions in buffered (pH > 7) aqueous media,^39^ including our 2013 report.^40^ In 2015, we demonstrated the application of orthogonal thiol–yne Michael and inverse electron-demand Diels–Alder additions in PBS (pH = 7.4) to afford dual-network hydrogels with outstanding strength (compressive stresses of 14 to 15 MPa at 98% compression). Importantly, both reactions were bioorthogonal, which allowed for the encapsulation of cells within the hydrogel matrix when the synthesis was conducted in cell culture media.

**Figure 7:**
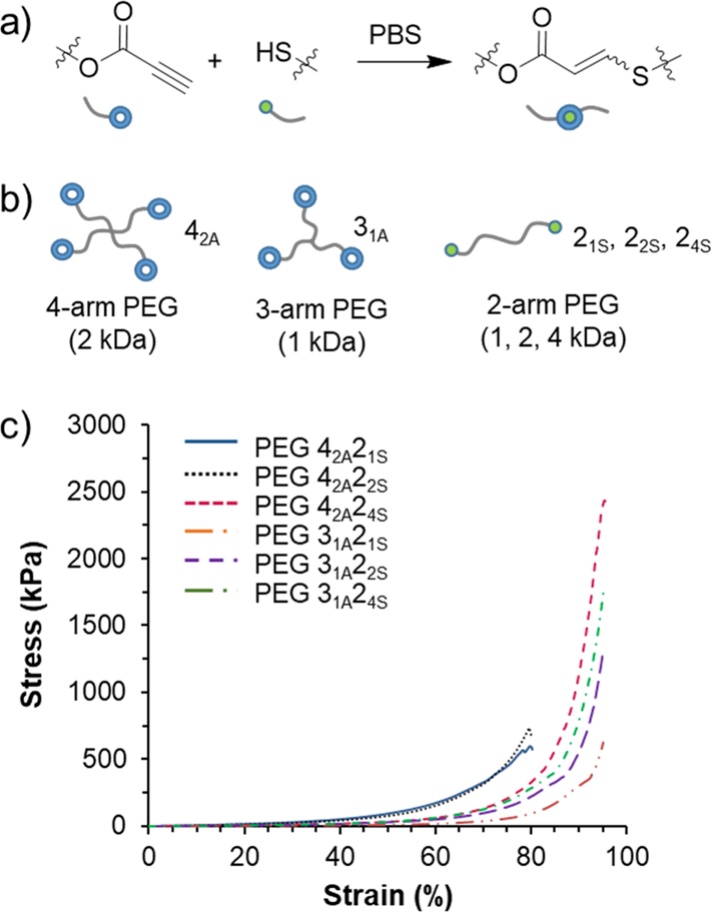
(a) Nucleophilic thiol–yne addition schematic. (b) Schematic of the PEG hydrogel precursors with notation. (Gels were formed from mixtures of alkyne and thiol precursors as indicated.) (c) Stress vs strain data for the compression analysis of hydrogels. Adapted with permission from ref (58). Copyright 2017 American Chemical Society.

In order to simplify the design of robust hydrogels, we thoroughly examined single network materials that were based solely on nucleophilic thiol–yne cross-linking.^58^ A library of gels were constructed from combinations of multi-armed (two-armed vs three-armed vs four-armed) PEG-modified propiolates and thiols which were synthesized using acid-catalyzed esterification reactions from commercial reagents (Figure 7). The reaction of oligomeric propiolates and thiols in PBS afforded tough, flexible hydrogels as evidenced by no change to their storage modulus, as determined by rheological measurements, for up to 100% strain. However, the storage modulus was able to be controlled over 3 orders of magnitude (0.18–7 MPa) depending on the topology (three-arm vs four-arm) and the molecular weight of the precursors (1 kDa vs 4 kDa), which we hypothesize influences the topology and density of the network formed. Notably, a hydrogel composed of a 2 kDa four-armed propiolate and a 4 kDa two-armed thiol (PEG4_2A_2_4S_) displayed the highest compressive strength at 2.4 MPa. This value is particularly striking considering that the gels contained 10% solids content or 90% water and featured only a single network. We attribute this to the high efficiency and rapid nature of the coupling reaction enabling the formation of a highly controlled network with minimal loops or defects. A later study on PEG4_2A_2_4S_ thiol–yne hydrogels greatly advanced the functionality of the materials.

In another study, the degradation rate and swellability of the PEG thiol–yne hydrogels were effectively controlled.^59^ While this was previously achieved by Truong et al. through replacing the PEG with a thermally responsive pluronic linker,^60^ we chose to additionally combine three- and/or four-armed PEG precursor substrates to balance the swellability that results from the hydrophilic contribution of the PEG backbone through increasing the number of cross-linking sites in the network, as demonstrated by Kamata and co-workers in other hydrogel systems.^61,62^ This afforded hydrogels which, after an initial shrinkage, remained largely unswollen over at least 30 days, which improved their mechanical resilience and allowed for tunable degradation rates. By extending this concept to allow for network functionalization in order to permit encapsulated cells to remodel the network, we also examined the limited introduction of two-arm dithiol precursors into a 3 + 3 network.^63^ Following this development, a two-arm matrix metalloproteinase (MMP) degradable dithiol-functionalized linker was incorporated into the network. This study was also focused on the addition of CGRGDS proteins to increase the cell interactions with the synthetic matrix and promote cell viability, achieved through thiol–yne attachment from the cysteine residue with an off-stoichiometry gel. This adjustment provided an unexpected advantage by removing the initial shrinkage phase, most likely a result of a slight loosening of the network. As may be anticipated, the incorporation of peptide fragments further influenced the swelling profiles because of their hydrophilicity.

**Figure 8:**
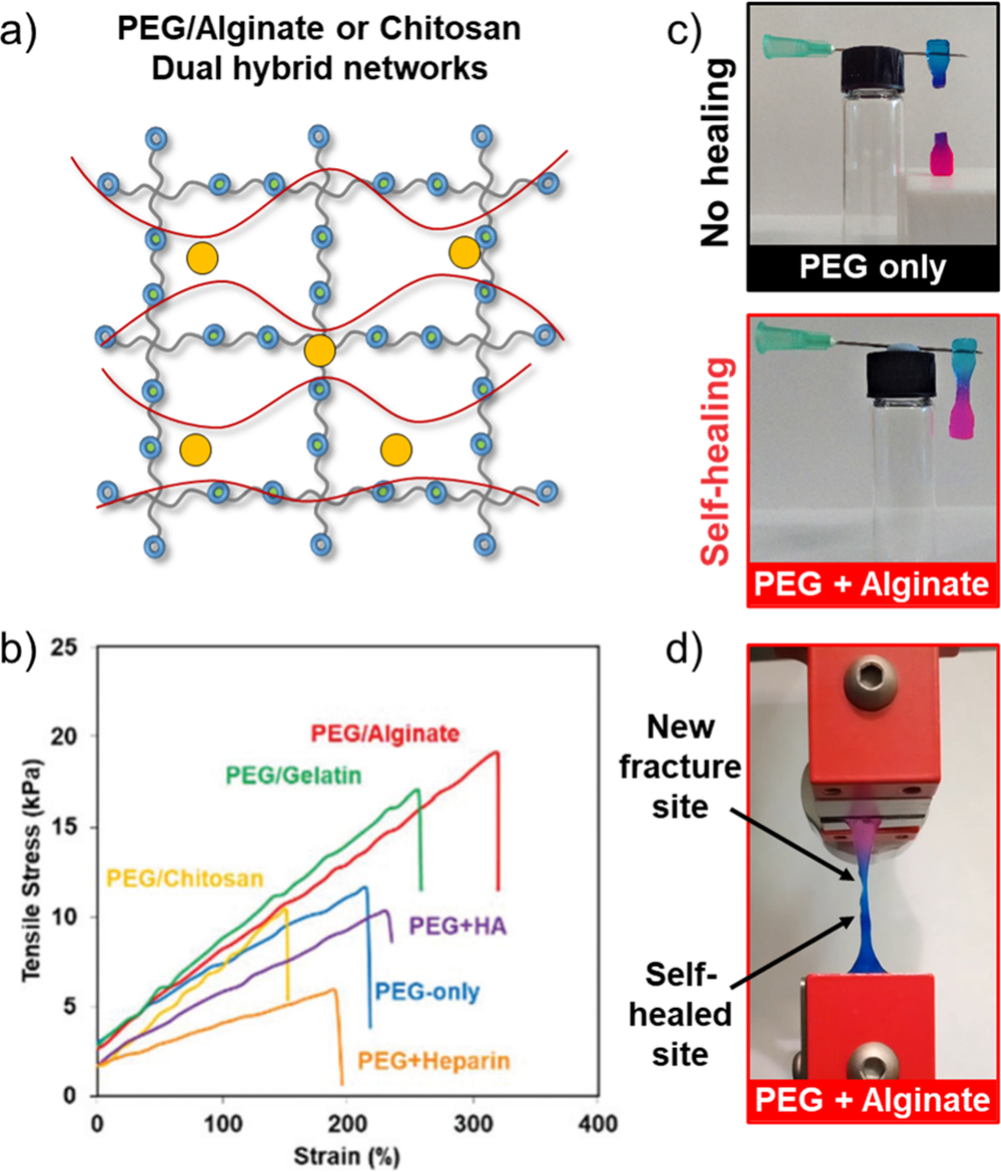
(a) Schematic of an interpenetrating hydrogel prepared by introducing a secondary loose network based on electrostatically cross-linked natural polymers (*i.e.*, alginate/calcium) and nucleophilic thiol–yne addition. (b) Representative tensile stress vs strain curve for PEG/natural polymer hydrogels. (c) Photographs of the self-healed PEG/alginate hydrogel against a PEG-only control. (d) Photograph of a re-healed PEG/alginate hydrogel undergoing tensile testing showing that the fracture site is not at the healed site. Adapted with permission from ref (64). Copyright 2018 The Authors. Published by Royal Society of Chemistry under the Creative Commons Attribution 3.0 Unported License (CC BY 3.0).

As an alternative method to increase hydrogel/cell interactivity in 3D, as well as to increase the sustainable content of the hydrogel materials, we created gels with interpenetrating hybrid networks formed from both a covalent thiol–yne PEG network and a noncovalent natural polysaccharide additive/network.^64^ Most notably, when the noncovalent network was formed from Ca/alginate, the hydrogels were less stiff (lower storage modulus) but had greater extensibility and ultimate tensile strength than the PEG-only single-network materials (Figure 8). Moreover, the ionic nature of the Ca/alginate network led to the observation of self-healing properties in the networks.

**Figure 9:**
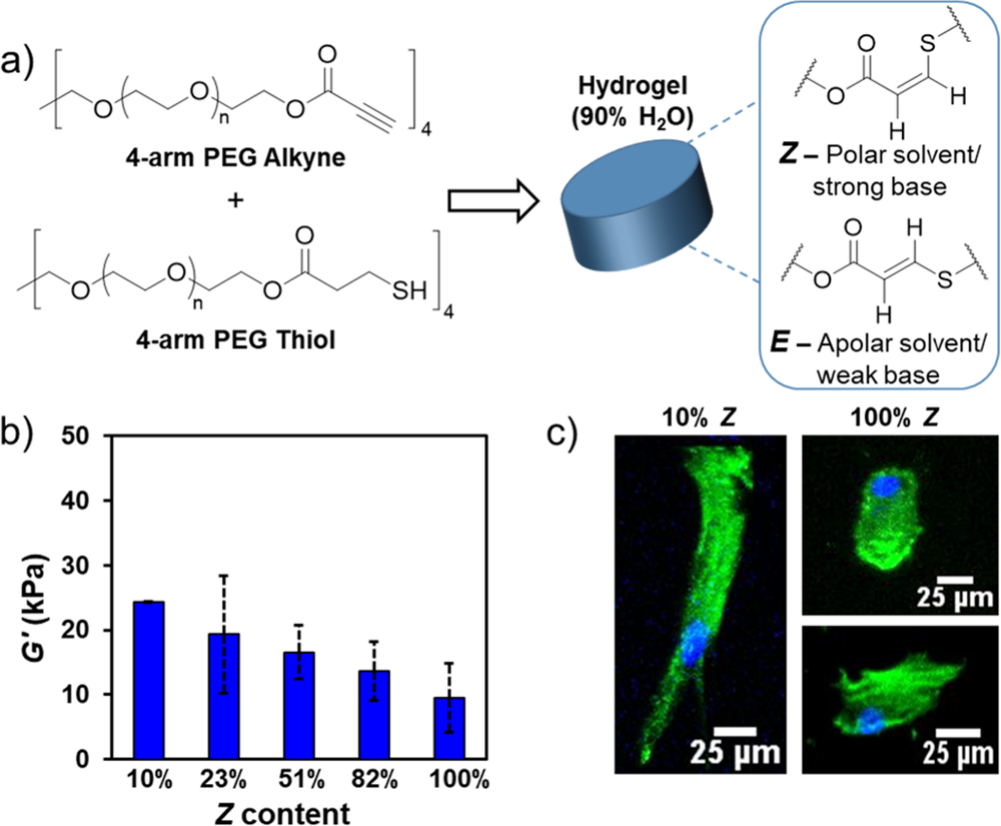
(a) Synthesis of thiol–yne click-hydrogels with controllable alkene stereochemistry by adjusting the reaction parameters. (b) Stiffness of hydrogels defined as the storage modulus (*G*′) at 0.1% strain. (c) Cell morphology of Y201 MSCs seeded on stereocontrolled hydrogels, assessed using phalloidin and DAPI staining following 72 h of culture. Adapted with permission from ref (3). Copyright 2021 The Authors. Published by Wiley under the Creative Commons Attribution 4.0 International (CC BY 4.0) License.

In a 2021 report, we adapted our method for the stereocontrolled thiol–yne reaction^40^ to synthesize hydrogels with tunable *E*/*Z*-alkene content to study cell mechanotransduction (Figure 9).^3^ Since the *E*/*Z* stereochemistry is dependent upon solvent polarity, we opted to use NEt_3_ as a base catalyst but varied the solvent environment during network synthesis. Hydrogels with nearly 100% *Z*-alkene stereochemistry were obtained in PBS, and high-*E* organogels (10% *Z*) were obtained from chloroform. Acetone, which is miscible with both chloroform and water, was used as an intermediate-polarity solvent to access organogels with mixed stereochemistry (23, 51, and 82% *Z*-content). Importantly, the gel fraction values were greater than 90% for all samples, which indicates a similar fundamental gel structure among formulations. The mesh size of the gels was also very consistent and ranged only from 4.5 nm (10% *E*) to 6.7 nm (100% *E*).

The organogels were also easily transformed into hydrogels via gradual solvent switching in PBS. This afforded robust scaffolds that exhibited minimal swellability, which was also highly consistent among all formulations. Despite their similar physical properties, the stereochemistry of the gel dictated its mechanical properties. Most significantly, the hydrogels with high *E*-content (10% *Z*) were approximately 3 times stiffer than gels with high *Z*-content (up to 100% *Z*). This enabled them to be studied as substrate stiffness reporters, in which the stiffness of the hydrogel could be decoupled from the chemical and physical aspects of the material, allowing us to study cell mechanotransduction without influences from other external factors.

## 4 Thiol–ene Polymers from Stereochemically Defined Monomers

Our stereoselective nucleophilic thiol–yne polymerizations afforded unsaturated polymers with approximately 30–80% *Z*-content,^1,2^ yet full stereocontrol remained elusive. We implemented a different strategy to obtain stereopure (100% *E* or Z) unsaturated polymers by employing monomers with predefined stereochemistry in conjunction with thiol–ene Michael addition. Previous work on creating unsaturated polymers from stereopure monomers tended to rely on using inefficient, high-temperature polycondensations, which can result in *Z* to *E* isomerization.^65,66^ In turn, this led to imperfect control over polymer properties, such as crystallinity, and the obfuscation of structure–property relationships. Milder, isomerization-free polycondensations of maleic anhydride yielded only low-molecular-weight polymers (*M*_w_ < 25 kDa).^67−69^ A chain-growth ring-opening polymerization of maleic anhydride afforded *Z*-isomer polyesters that were also thermally isomerized to *E*-isomers, but again, typically the molecular weights of the polymers that resulted were still limited to *M*_w_ < 30 kDa.^70^

**Figure 10:**
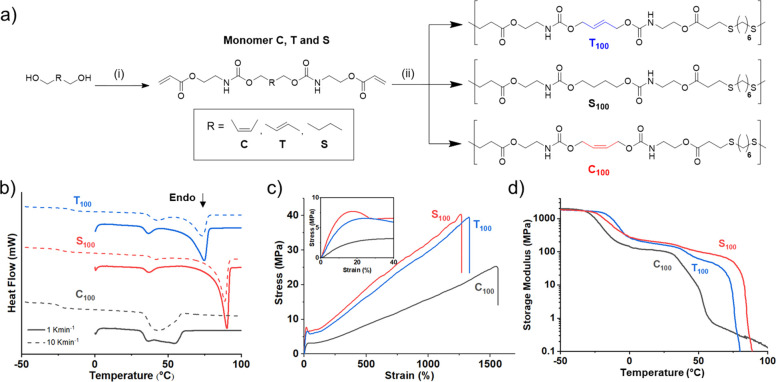
(a) Synthesis of copolymers with stereochemically defined double bonds using the thiol–ene Michael addition of dithiols and diacrylate monomers. (i) 2.1 equiv of 2-isocyanatoethyl acrylate, 0.2 mol % of dibutyl tin(IV) dilaurate, THF, 22 °C; (ii) 1 equiv of 1,6-hexanedithiol, 2 mol % dimethylphenylphosphine (DMPP), DMF, 22 °C. (b) DSC thermograms of homopolymers for the first heating cycle (dashed lines = 10 K·min^–1^ and solid lines = 1 K·min^–1^). (c) Respresentative stress vs strain curves of homopolymers (*n* = 5). Inset data between 0 and 40% strain. (d) Dynamic mechanical thermal analysis thermograms of storage modulus vs temperature performed in the tensile configuration. Polymer data: T_100_, *M*_w_ = 103 kDa and *Đ*_M_ = 3.57; S_100_, *M*_w_ = 139 kDa and *Đ*_M_ = 4.51; C_100_, *M*_w_ = 250 kDa and *Đ*_M_ = 7.19. Adapted with permission from ref (71). Copyright 2020 American Chemical Society.

We devised a one-step method to afford stereopure alkene-containing diacrylate monomers, which were suitable for a mild thiol–ene click polymerization (Figure 10).^71^ We selected acrylates as Michael acceptors, rather than the more active maleimides,^72,73^ due to a 2018 study by Long and co-workers that showed that high-molar-mass polyesters (up to 70 kDa *M*_w_) could be obtained from the nucleophilic thiol–ene step-growth polymerization of acrylates.^74^ The absolute control of alkene stereochemistry (0–100% *E*-content) in the polymer backbone was demonstrated by modulating the *E*/*Z* double-bond content with the monomer feed. Undesirable isomerization was mitigated, especially for the high-*Z* polymers (C_100_), as a result of the mild conditions under which the click polymerization was conducted. High-molecular-weight (*M*_w_ = 103–250 kDa) poly(ester-urethanes) were isolated after reacting for 2 h at ambient temperature (Figure 10a). Dimethylphenylphoshine (DMPP) was found to be a highly active polymerization catalyst, as previously shown by Lowe and Bowman in small-molecule studies,^75^ but we have also found DBU to have a similar level of activity in related thiol–ene polymerizations.

Moreover, we designed monomers that featured internal urethane moieties by reacting alcohol precursors with commercially available 2-isocyanatoethyl acrylate (Figure 10a). We anticipated that the poly(ester-urethane)s would display enhanced mechanical properties as compared to all polyester variants (synthesized only from acrylates) due to strong hydrogen-bonding interactions of the urethanes. This key design feature would again be exploited in some of our later studies. Both 100% *E-* and 100% *Z*-polymers (T_100_ and C_100_) as well as a saturated analogue (S100) were all semicrystalline thermoplastics with good mechanical properties. The *E*polymer and saturated polymer displayed sharp melt transitions at 75 and 90 °C, respectively (Figure 10b). However, the *Z-*alkene polymer possessed a complex melt profile centered around 50 °C, in addition to a decreased total enthalpy of melting (Δ*H*_m_). These thermal differences were manifested in their mechanical properties where the *Z*-polymer was softer, weaker, but more extensible than either the saturated or *E*-polymer (Figure 10c,d). The simple polymerization method allowed us to precisely make polymers of mixed stereochemistry to access intermediate properties between the two stereopure samples. In a later study, we employed the same *E*- and *Z-*alkene monomers to synthesize stereopure telechelic oligomers with molar masses ranging from 4 to 10 kDa.^76^ Importantly, the oligomers possessed acrylate end-groups as the stoichiometry of the acrylate monomer to thiol was biased. The alkene-containing oligomers were then orthogonally cross-linked into networks *via* acrylate photopolymerization. The backbone alkenes were unreactive under these conditions, and equally, no photoisomerization was observed. Thus, we were able to formulate 3D-printed soft elastomers (*Z*-isomers) or stiff plastics (*E*-isomers) which also possessed degradation rates that differed according to stereochemistry.

We have also investigated click step-growth polymerizations for monomers possessing geometric isomerism that is induced by planar rigidity imparted by ring units. We employed a similar strategy to build polyester diastereomers from diacrylate *Z* or *E* cyclopropane monomers.^77^ Surprisingly, both *Z*- and *E*- polymers were amorphous with low *T*_g_’s (*Z* = −47 °C; *E* = −52 °C). In comparison, a polyester obtained from 1,4-butanediol (*i.e*., without a rigid ring in the backbone) was semicrystalline. Even though the isomeric cyclopropane polymers were both amorphous, the incorporation of a small number of cyclopropane units (*via* copolymerization) into a semicrystalline polymer afforded rational control of the bulk crystallinity, which had a knock-on effect on the mechanical properties, allowing for the manipulation of strength and stiffness.

Building on the cyclopropane work, we turned our attention to renewable cyclic building blocks. Rigid-ring bicyclic ethers that are produced from the dehydrative cyclization of sugar alcohols have been widely explored in polymer chemistry because of their unique structures, which offer planar geometric isomerism and inherent degradability.^78−80^ The most prevalent cyclic unit is the glucose-derived isosorbide, with the accompanying diastereomers, isomannide and isoidide, being less studied. Many isohexide-based polymers, with the exception of multiblock architectures, are brittle (high-*T*_g_) plastics as a consequence of the incorporation of the rigid ring in the polymer backbone.^78−81^ Some notable exceptions are low-*T*_g_ (below ambient temperature) thermoplastic elastomer polyethers^82^ or polyesters^83−85^ from Reineke and co-workers; however, the best material properties (*i.e.*, high strength and extensibility) were observed for copolymers that also incorporated a complementary dilactone monomer.

**Figure 11:**
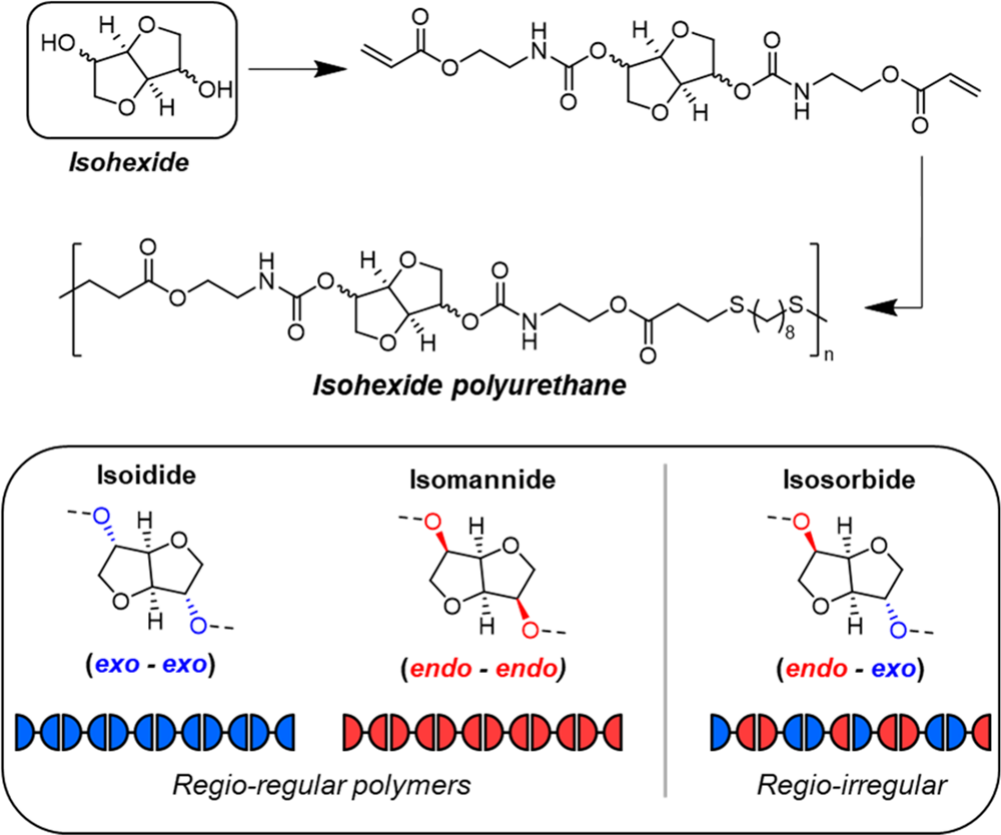
Synthesis of isohexide-containing polymers from isoidide (*exo*/*exo*), isomannide (*endo*/*endo*), and isosorbide (*endo*/*exo*) by thiol–ene nucleophilic addition. Adapted with permission from ref (4). Copyright 2022 American Chemical Society.

Nevertheless, these works by Reineke and co-workers inspired our attempts to build high-molecular-weight isohexide-containing polymers using the nucleophilic thiol–ene polymerization.^4,86^ Since each isohexide features fused tetrahydrofuranyl rings with two hydroxyl substituents in *endo* or *exo* conformations (isosorbide – *endo*/*exo*; isomannide – *endo*/*endo*; and isoidide – *exo*/*exo*), the same monomer strategy used to synthesize urethane-acrylate monomers from simple diols^71^ was conveniently applied to all three isohexide diastereomers. Thus, each monomer contained a rigid-bicyclic core (stereopure isohexide) with flanking internal urethane groups and reactive acrylate groups with which to build molar mass through phosphine-catalyzed thiol–ene step-growth polymerization (Figure 11). In each case, with a commercial dithiol, high-molecular-weight (typically *M*_w_ > 100 kDa) linear poly(ester-urethanes) were readily obtained (Figure 11).^4,86^ It was immediately clear that the stereochemistry of the isohexide was dictating the thermomechanical properties and physical behavior of the polymers. Molecular weight and/or dispersity differences were also ruled out as factors in producing the dissimilar performance among the polymers. Moreover, irrespective of stereochemistry, the isohexide polymers displayed outstanding mechanical performance which was beyond that of many leading commercial materials.

Both ISPU and IMPU polymers formed amorphous materials of high optical transparency that behaved as tough elastomers with high tensile strength (ISPU = 75 MPa; IMPU = 63.5 MPa) and elongation at break (ISPU = 1466%; IMPU = 1806%).^86^ These mechanical properties are beyond those of conventional thermoplastic elastomers and even covalently cross-linked rubbers. Furthermore, each elastomer featured a “J-shaped” tensile curve with three discrete elastic regimes and a significant strain-hardening phase in the third region. The stereochemistry of the isohexide also impacted the elastic recovery of each material. Across all three elastic regimes and regardless of the deformation rate, IMPU recovered more slowly than ISPU. Molecular dynamics simulations provided insight into these differences and revealed that this was driven by the evolution of the hydrogen-bonding network.

**Figure 12:**
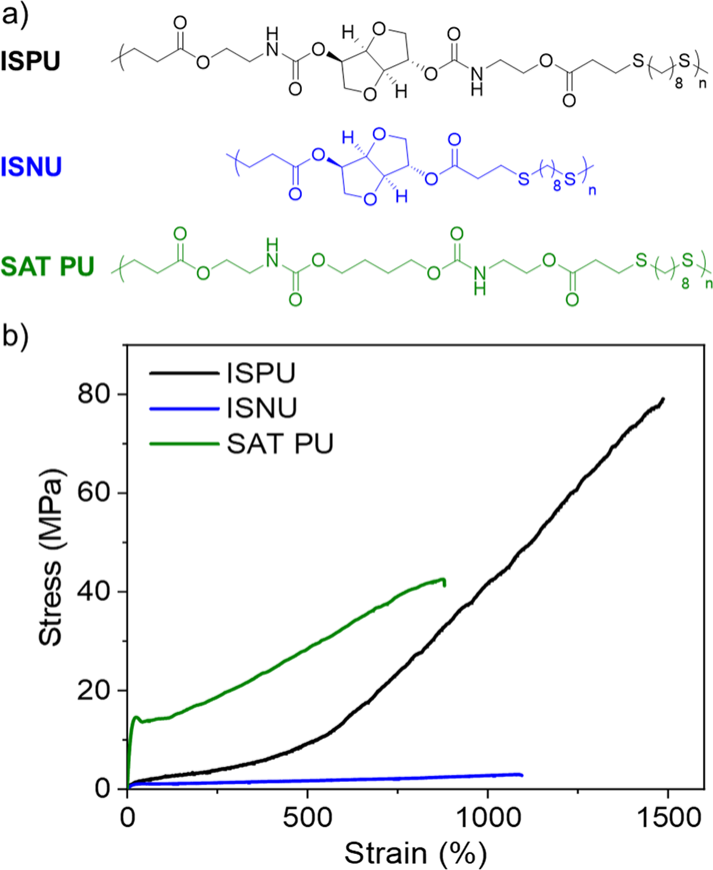
(a) Structures of ISPU (urethane and isosorbide), ISNU (isosorbide only), and SAT-PU (urethane only). (b) Stress vs strain curves obtained by tensile testing of ISPU, ISNU, and SAT-PU films. Polymer data: ISPU, *M*_w_ = 110 kDa and *Đ*_M_ = 11.08; ISNU, *M*_w_ = 136 kDa and *Đ*_M_ = 6.71; SAT PU, *M*_w_ = 139 kDa and *Đ*_M_ = 4.71. Adapted with permission from ref (86). Copyright 2022 The Authors. Published by Wiley under the Creative Commons Attribution 4.0 International (CC BY 4.0) License.

Importantly, X-ray scattering data revealed that the strain hardening was not due to strain-induced crystallization. Thus, we posited that the materials were transiently cross-linking by a dynamic hydrogen-bonding network, and this was confirmed by mechanical testing at different strain rates. To further understand the structural features that were responsible for the behavior, two additional polymers were synthesized and compared to ISPU to illustrate the importance of having both the urethane moiety and the rigid isohexide unit in the polymer backbone (Figure 12). When the isosorbide was replaced with 1,4-butanediol, the resultant polymer (SAT-PU) was a semicrystalline plastic with good tensile strength, though it was still noticeably weaker than ISPU. This finding was consistent with our previous study.^71^ Conversely, removal of the urethane linkage afforded an isosorbide-based polyester (ISNU) that was also semicrystalline yet much weaker (tensile strength of up to 10.8 MPa). Thus, the combination of the rigid sugar moiety with strong-hydrogen-bonding urethane was critical to the formation of extremely tough and elastic materials (*i.e.*, pronounced strain-hardening without crystallization).

**Figure 13:**
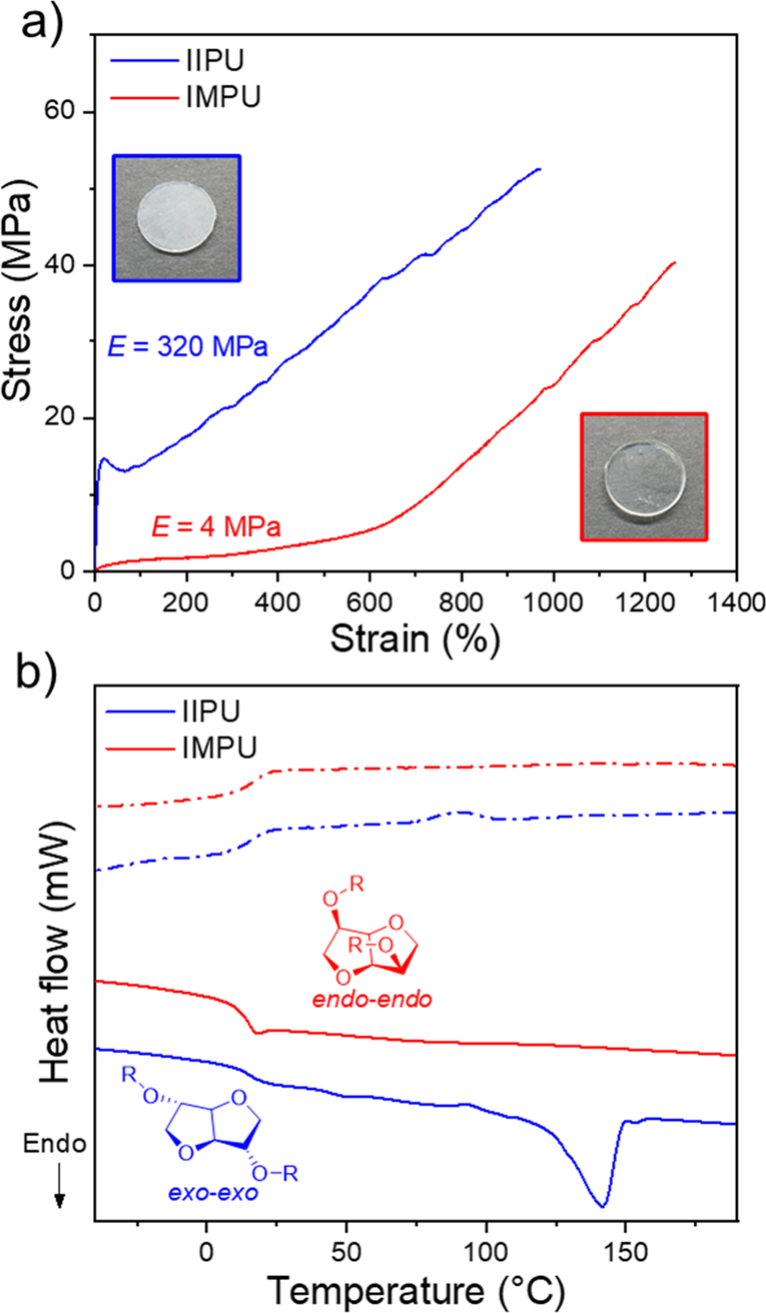
(a) Stress vs strain curves obtained by tensile testing of IIPU (isoidide polyurethane) and IMPU (isomannide polyurethane). (Inset) Photographs of pressed films of IIPU and IMPU. (b) DSC thermograms of the first heating and cooling cycle for IIPU and IMPU (solid line = heating scan and dashed line = cooling scan). Polymer data: IIPU, *M*_w_ = 117 kDa and *Đ*_M_ = 8.12; IMPU, *M*_w_ = 95 kDa and *Đ*_M_ = 9.69. Adapted with permission from ref (4). Copyright 2022 American Chemical Society.

In sharp contrast to the elastomeric nature of the isosorbide and isomannide isomers, the isoidide (*exo*/*exo*) polymer (IIPU) displayed distinctly different behavior with a clear yield point, which is a hallmark of plastic deformation behavior (Figure 13a). In fact, the stiffness (Young’s modulus = 320 MPa) and ductility (elongation at break near 1000%) of IIPU were comparable to those of commodity thermoplastics such as high-density polyethylene. However, IIPU also possessed a high tensile strength of 56 MPa, which is more similar to that of an engineering plastic such as nylon. The thermal properties of IIPU (Figure 13b) showed it to be semicrystalline (*T*_m_ = 140 °C). A prevailing dogma in polymer design is that stereochemistry can incrementally change properties (*i.e.*, make a weak plastic stronger, stiffer, or more ductile) but cannot change baseline behavior. However, here opposing mechanical behavior (elastic vs plastic) was observed between isomeric polymers. To shed light on this behavior, we again turned to molecular dynamic simulations to model hydrogen-bonding interactions under deformation. IIPU was found to possess about 50% more total hydrogen bonds than IMPU. IIPU chains also revealed a preference to favor intermolecular hydrogen bonds. Together, these observations may suggest a pathway by which IIPU samples can crystallize. Thus, the opposing material behavior was manifested by acute differences in hydrogen bonding (intra- vs interchain interactions), which was driven by the stereochemistry of the isohexide.

**Figure 14:**
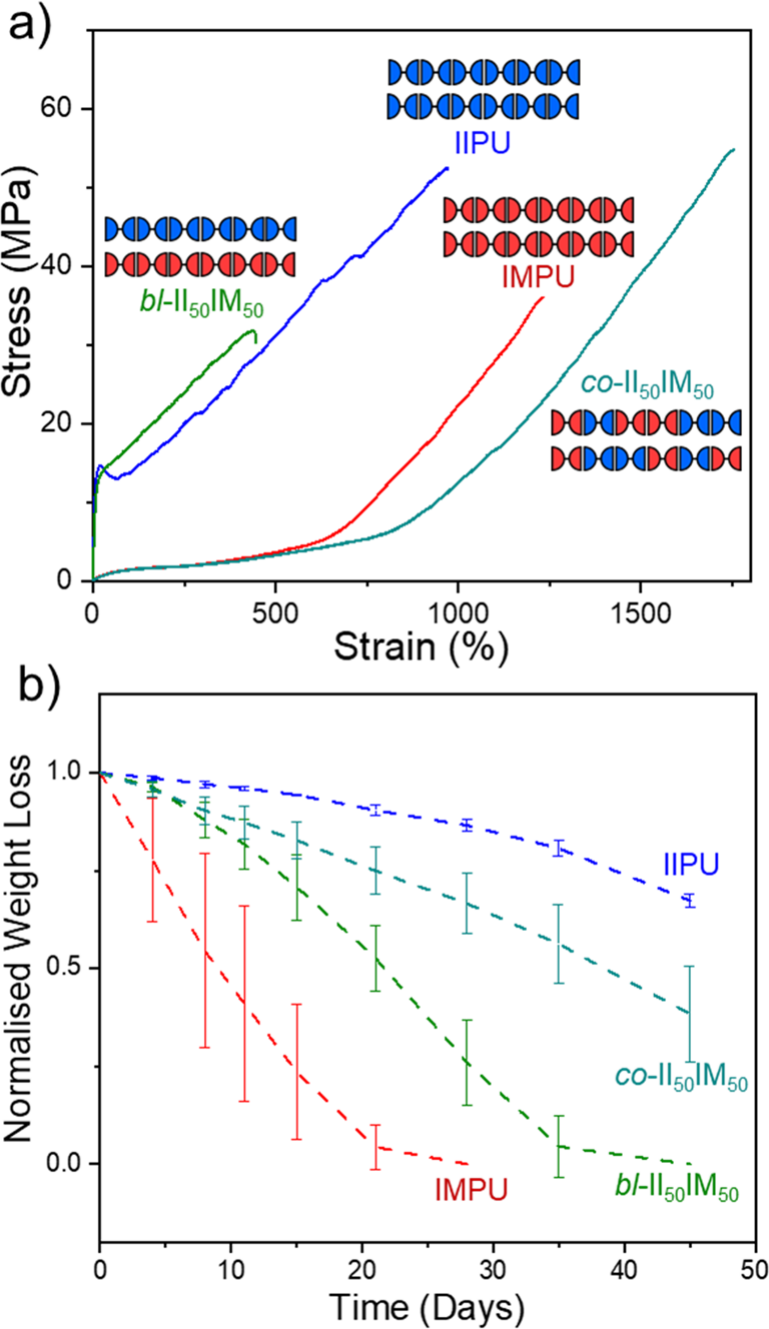
(a) Stress vs strain tensile curves of annealed IIPU, IMPU, and both a statistical copolymer (II_50_IM_50_) and a physical blend (*bl*-II_50_IM_50_) at 50/50 II/IM. (b) Normalized weight loss of IIPU, IMPU, *co*-II_50_IM_50_, and *bl*-II_50_IM_50_ discs in 1 M NaOH(aq) over 45 days at 25 °C. Polymer data: IIPU, *M*_w_ = 117 kDa and *Đ*_M_ = 8.12; IMPU, *M*_w_ = 95 kDa and *Đ*_M_ = 9.69; *co*-II_50_IM_50_, *M*_w_ = 79 kDa and *Đ*_M_ = 7.81; *bl*-II_50_IM_50_, *M*_w_ = 85 kDa and *Đ*_M_ = 4.82. Adapted with permission from ref (4). Copyright 2022 American Chemical Society.

Since IMPU and IIPU were produced from isomeric monomers, isoidide and isomannide were easily co-incorporated into materials either as statistical copolymers or from blended homopolymers despite their contrasting thermomechanical properties. The consequence of this exceptional compatibility was significant as it allowed for independent tuning, or decoupling, of the degradation rate from mechanical properties (Figure 14a,b). This simple strategy demonstrates a viable path to materials with on-demand property tuning *via* stereochemical manipulation. These results were also striking since the degradation rates did not follow crystallinity trends. Despite the blend and copolymer materials having the same composition (50/50 isoidide/isomannide), the blended sample was fast-degrading yet semicrystalline while the copolymer was relatively slow-degrading yet amorphous. We observed evidence of phase separation using atomic force microscopy (AFM), which was backed up by molecular dynamics simulations, to reveal that the blended sample was less homogeneous than the copolymer and possessed larger domains of isoidide- and isomannide-rich regions, likely contributing to differences in their respective degradation rates. In effect, the phase separation and larger IIPU domains likely facilitated crystallization and contributed to the plastic behavior of the blended sample. Despite their phase separation and enhanced degradability, the blended samples were strong plastics (even for other ratios of IIPU and IMPU), signaling potential application for mechanical recycling of mixed polymer feeds.

## 5 Summary and Outlook

Click step-growth polymerization methods are attractive and, increasingly, practical alternatives to conventional polycondensation reactions. In particular, the thiol–yne/–ene Michael reaction is a straightforward technique for producing high-molecular-weight step-growth polymers in an exceedingly efficient manner. The reactions can be conducted at ambient temperature, in open air, and using only catalytic amounts of common amines or phosphines. Beyond the green reaction metrics, the reactions provide access to polymeric structures of diverse composition or functionality (including polyesters and polyamides, among others) due to the accessibility of different Michael acceptor monomers and commercial dithiols combined with excellent functional group tolerance.

Click step-growth polymerizations can also be effectively used to create stereocontrolled polymer architectures, many of which are unfeasible to attain when using conventional polycondensation methods. With simple reaction modifications, the thiol–yne Michael polymerization produces materials with well-defined *E*/*Z*-isomerism (*ca*. 30–80% *Z*-content) along the polymer backbone. On the other hand, the thiol–ene Michael polymerization can be used to produce stereopure (100% *E* or *Z*) unsaturated polymers from monomers with predefined stereochemistry. Many of the polymers (and hydrogels) that we showcased in this Account exhibit outstanding mechanical performance, from strong plastics to tough elastomers, which can also be exquisitely tailored according to the stereochemistry of the polymers. Due to the exceptional compatibility of the isomeric monomers and polymers, there are opportunities to create a large range of materials from a small monomer pool *via* simple copolymerization or blending strategies.

Combining the use of renewable feedstocks with efficient synthetic methods and green chemistry principles^87^ is imperative to achieving truly sustainable polymers with minimal environmental impact. However, bringing together green chemistry methodology and sustainable starting materials without compromising performance is an enduring challenge in polymer synthesis. Yet in this Account, we have demonstrated that renewable diols can be efficiently transformed, often in one synthetic step, into highly active monomers for thiol–yne/–ene click polymerization. The resultant polymers that contain degradable motifs displayed uncompromised mechanical performance that makes them competitive with, or even superior to, many existing commodity plastics and elastomers.

Despite our contributions and other advances in the field, we propose several innovation areas to unlock the full potential of thiol–yne/–ene click polymerizations and click polymerizations more generally:

I. Increase the stereoselectivity of the thiol–yne reaction to further tailor polymer performance.
II. Replace chlorinated polymerization solvents with greener alternative solvents or processes.
III. Identify additional sustainable monomer feedstock to replace non-sustainably derived components in the polymers, including dithiols, urethanes, and acrylates.
IV. Improve the microstructural control of the polymer backbone, such as sequence-specific stereoblocks, to reclaim advantages associated with chain-growth polymerization methods.
V. Design closed-loop chemically recyclable click polymers.

## References

[ref1] Bell C. A.; Yu J.; Barker I. A.; Truong V. X.; Cao Z.; Dobrinyin A. V.; Becker M. L.; Dove A. P. Independent Control of Elastomer Properties through Stereocontrolled Synthesis. Angew. Chem. Int. Ed. 2016, 55, 13076–13080. 10.1002/anie.201606750. PMC5082520 27654023

[ref2] Worch J. C.; Weems A. C.; Yu J.; Arno M. C.; Wilks T. R.; Huckstepp R. T. R.; O’Reilly R. K.; Becker M. L.; Dove A. P. Elastomeric polyamide biomaterials with stereochemically tuneable mechanical properties and shape memory. Nat. Commun. 2020, 11, 3250 10.1038/s41467-020-16945-8. 32591525 PMC7320000

[ref3] Macdougall L. J.; Pérez-Madrigal M. M.; Shaw J. E.; Worch J. C.; Sammon C.; Richardson S. M.; Dove A. P. Using Stereochemistry to Control Mechanical Properties in Thiol–Yne Click-Hydrogels. Angew. Chem. Int. Ed. 2021, 60, 25856–25864. 10.1002/anie.202107161. PMC9298389 34551190

[ref4] Stubbs C. J.; Worch J. C.; Prydderch H.; Wang Z.; Mathers R. T.; Dobrynin A. V.; Becker M. L.; Dove A. P. Sugar-Based Polymers with Stereochemistry-Dependent Degradability and Mechanical Properties. J. Am. Chem. Soc. 2022, 144, 1243–1250. 10.1021/jacs.1c10278. 35029980 PMC8796236

[ref5] Geng Z.; Shin J. J.; Xi Y.; Hawker C. J. Click chemistry strategies for the accelerated synthesis of functional macromolecules. J. Polym. Sci. 2021, 59, 963–1042. 10.1002/pol.20210126.

[ref6] Billiet L.; Hillewaere X. K. D.; Du Prez F. E. Highly functionalized, aliphatic polyamides via CuAAC and thiol-yne chemistries. Eur. Polym. J. 2012, 48, 2085–2096. 10.1016/j.eurpolymj.2012.08.013.

[ref7] Besset C.; Binauld S.; Ibert M.; Fuertes P.; Pascault J.-P.; Fleury E.; Bernard J.; Drockenmuller E. Copper-Catalyzed vs Thermal Step Growth Polymerization of Starch-Derived α-Azide−ω-Alkyne Dianhydrohexitol Stereoisomers: To Click or Not To Click?. Macromolecules 2010, 43, 17–19. 10.1021/ma9024784.

[ref8] Binauld S.; Boisson F.; Hamaide T.; Pascault J.-P.; Drockenmuller E.; Fleury E. Kinetic study of copper(I)-catalyzed Click chemistry step-growth polymerization. J. Polym. Sci., Part A: Polym. Chem. 2008, 46, 5506–5517. 10.1002/pola.22871.

[ref9] Song H. B.; Baranek A.; Bowman C. N. Kinetics of bulk photo-initiated copper(I)-catalyzed azide–alkyne cycloaddition (CuAAC) polymerizations. Polym. Chem. 2016, 7, 603–612. 10.1039/C5PY01655J. 27429650 PMC4946250

[ref10] Brändle A.; Khan A. Thiol–epoxy ‘click’ polymerization: Efficient construction of reactive and functional polymers. Polym. Chem. 2012, 3, 3224–3227. 10.1039/c2py20591b.

[ref11] Binder S.; Gadwal I.; Bielmann A.; Khan A. Thiol-epoxy polymerization via an AB monomer: Synthetic access to high molecular weight poly(β-hydroxythio-ether)s. J. Polym. Sci., Part A: Polym. Chem. 2014, 52, 2040–2046. 10.1002/pola.27212.

[ref12] Dong J.; Sharpless K. B.; Kwisnek L.; Oakdale J. S.; Fokin V. V. SuFEx-based synthesis of polysulfates. Angew. Chem. Int. Ed. 2014, 53, 9466–9470. 10.1002/anie.201403758. PMC4442796 25100330

[ref13] Barrow A. S.; Smedley C. J.; Zheng Q.; Li S.; Dong J.; Moses J. E. The growing applications of SuFEx click chemistry. Chem. Soc. Rev. 2019, 48, 4731–4758. 10.1039/C8CS00960K. 31364998

[ref14] Zhao T.; Beyer V. P.; Becer C. R. Fluorinated polymers via para-fluoro-thiol and thiol-bromo Click step growth polymerization. Macromol. Rapid Commun. 2020, 41, 2000409 10.1002/marc.202000409. 32989854

[ref15] Park N. H.; Gomes G. d. P.; Fevre M.; Jones G. O.; Alabugin I. V.; Hedrick J. L. Organocatalyzed synthesis of fluorinated poly(aryl thioethers). Nat. Commun. 2017, 8, 166 10.1038/s41467-017-00186-3. 28761127 PMC5537313

[ref16] Collins J.; Xiao Z.; Espinosa-Gomez A.; Fors B. P.; Connal L. A. Extremely rapid and versatile synthesis of high molecular weight step growth polymers via oxime click chemistry. Polym. Chem. 2016, 7, 2581–2588. 10.1039/C6PY00372A.

[ref17] Hoyle C. E.; Bowman C. N. Thiol–Ene Click Chemistry. Angew. Chem. Int. Ed. 2010, 49, 1540–1573. 10.1002/anie.200903924. 20166107

[ref18] Lowe A. B.; Hoyle C. E.; Bowman C. N. Thiol-yne Click Chemistry: A Powerful and Versatile Methodology for Materials Synthesis. J. Mater. Chem. 2010, 20, 4745–4750. 10.1039/b917102a.

[ref19] Fairbanks B. D.; Scott T. F.; Kloxin C. J.; Anseth K. S.; Bowman C. N. Thiol–Yne Photopolymerizations: Novel Mechanism, Kinetics, and Step-Growth Formation of Highly Cross-Linked Networks. Macromolecules 2009, 42, 211–217. 10.1021/ma801903w. 19461871 PMC2651690

[ref20] Hoogenboom R. Thiol–Yne Chemistry: A Powerful Tool for Creating Highly Functional Materials. Angew. Chem. Int. Ed. 2010, 49, 3415–3417. 10.1002/anie.201000401. 20394091

[ref21] Türünç O.; Meier M. A. R. A novel polymerization approach via thiol-yne addition. J. Polym. Sci., Part A: Polym. Chem. 2012, 50, 1689–1695. 10.1002/pola.25957.

[ref22] Hensarling R. M.; Doughty V. A.; Chan J. W.; Patton D. L. “Clicking” Polymer Brushes with Thiol-yne Chemistry: Indoors and Out. J. Am. Chem. Soc. 2009, 131, 14673–14675. 10.1021/ja9071157. 19778016

[ref23] Koo S. P. S.; Stamenović M. M.; Prasath R. A.; Inglis A. J.; Du Prez F. E.; Barner-Kowollik C.; Van Camp W.; Junker T. Limitations of radical thiol-ene reactions for polymer–polymer conjugation. J. Polym. Sci., Part A: Polym. Chem. 2010, 48, 1699–1713. 10.1002/pola.23933.

[ref24] Griesbaum K. Problems and Possibilities of the Free-Radical Addition of Thiols to Unsaturated Compounds. Angew. Chem. Int. Ed. 1970, 9, 273–287. 10.1002/anie.197002731.

[ref25] Stanford M. J.; Pflughaupt R. L.; Dove A. P. Synthesis of Stereoregular Cyclic Poly(lactide)s via “Thiol–Ene” Click Chemistry. Macromolecules 2010, 43, 6538–6541. 10.1021/ma101291v.

[ref26] Pounder R. J.; Stanford M. J.; Brooks P.; Richards S. P.; Dove A. P. Metal free thiol–maleimide ‘Click’ reaction as a mild functionalisation strategy for degradable polymers. Chem. Commun. 2008, 5158–5160. 10.1039/b809167f. 18956054

[ref27] Hall D. J.; Van Den Berghe H. M.; Dove A. P. Synthesis and post-polymerization modification of maleimide-containing polymers by ‘thiol-ene’ click and Diels–Alder chemistries. Polym. Int. 2011, 60, 1149–1157. 10.1002/pi.3121.

[ref28] Billiet L.; Gok O.; Dove A. P.; Sanyal A.; Nguyen L.-T. T.; Du Prez F. E. Metal-Free Functionalization of Linear Polyurethanes by Thiol-Maleimide Coupling Reactions. Macromolecules 2011, 44, 7874–7878. 10.1021/ma201323g.

[ref29] Onbulak S.; Tempelaar S.; Pounder R. J.; Gok O.; Sanyal R.; Dove A. P.; Sanyal A. Synthesis and Functionalization of Thiol-Reactive Biodegradable Polymers. Macromolecules 2012, 45, 1715–1722. 10.1021/ma2019528.

[ref30] Ruhemann S.; Stapleton H. E. CIX.—Condensation of Phenols with Esters of the Acetylene Series. Part III. Synthesis of Benzo-γ-pyrone. J. Chem. Soc., Trans. 1900, 77, 1179–1185. 10.1039/CT9007701179.

[ref31] Truce W. E.; Simms J. A. Stereospecific Reactions of Nucleophilic Agents with Acetylenes and Vinyl-type Halides. IV. The Stereochemistry of Nucleophilic Additions of Thiols to Acetylenic Hydrocarbons^1^. J. Am. Chem. Soc. 1956, 78, 2756–2759. 10.1021/ja01593a029.

[ref32] Truce W. E.; Heine R. F. The Stereochemistry of Base-catalyzed Additions of *p*-Toluenethiol to Several Negatively-substituted Acetylenes. An Exception to the Rule of *Trans*-nucleophilic Addition^1, 2^. J. Am. Chem. Soc. 1957, 79, 5311–5313. 10.1021/ja01576a062.

[ref33] Truce W. E.; Goldhamer D. L. The Stereochemistry of the Base-catalyzed Addition of *p*-Toluenethiol to Sodium and Ethyl Phenylpropiolate^1,2^. J. Am. Chem. Soc. 1959, 81, 5795–5798. 10.1021/ja01530a060.

[ref34] Truce W. E.; Tichenor G. J. W. Effect of Activating Group on *Trans*-stereoselectivity of Thiolate Additions to Activated Acetylenes. J. Org. Chem. 1972, 37, 2391–2396. 10.1021/jo00980a007.

[ref35] Tawfik O. M.; Nabih B. M. Stereochemistry of Ionic Thiol Addition to Acetylenic Ketones. Bull. Chem. Soc. Jpn. 1974, 47, 2325–2326. 10.1246/bcsj.47.2325.

[ref36] Arjona O.; Medel R. o.; Rojas J.; Costa A. M.; Vilarrasa J. Chemoselective protection of thiols versus alcohols and phenols. The Tosvinyl group. Tetrahedron Lett. 2003, 44, 6369–6373. 10.1016/S0040-4039(03)01614-9.

[ref37] Crisp G. T.; Millan M. J. Conjugate addition of amino acid side chains to alkynones and alkynoic acid derivatives. Tetrahedron 1998, 54, 637–648. 10.1016/S0040-4020(97)10323-4.

[ref38] Kondoh A.; Takami K.; Yorimitsu H.; Oshima K. Stereoselective Hydrothiolation of Alkynes Catalyzed by Cesium Base: Facile Access to (*Z*)-1-Alkenyl Sulfides. J. Org. Chem. 2005, 70, 6468–6473. 10.1021/jo050931z. 16050711

[ref39] Shiu H.-Y.; Chan T.-C.; Ho C.-M.; Liu Y.; Wong M.-K.; Che C.-M. Electron-Deficient Alkynes as Cleavable Reagents for the Modification of Cysteine-Containing Peptides in Aqueous Medium. Chem.—Eur. J. 2009, 15, 3839–3850. 10.1002/chem.200800669. 19229937

[ref40] Truong V. X.; Dove A. P. Organocatalytic, Regioselective Nucleophilic “Click” Addition of Thiols to Propiolic Acid Esters for Polymer–Polymer Coupling. Angew. Chem. Int. Ed. 2013, 52, 4132–4136. 10.1002/anie.201209239. 23450768

[ref41] Worch J. C.; Prydderch H.; Jimaja S.; Bexis P.; Becker M. L.; Dove A. P. Stereochemical enhancement of polymer properties. Nat. Rev. Chem. 2019, 3, 514–535. 10.1038/s41570-019-0117-z.

[ref42] Bunn C. W. Molecular Structure and Rubber-like Elasticity I. The Crystal Structures of β Gutta-Percha, Rubber and Polychloroprene. Proc. R. Soc. London A 1942, 180, 40–66. 10.1098/rspa.1942.0024.

[ref43] Bunn C. W. Molecular Structure and Rubber-like Elasticity III. Molecular Movements in Rubber-like Polymers. Proc. R. Soc. London A 1942, 180, 82–99. 10.1098/rspa.1942.0026.

[ref44] Tanaka R.; Yuuya K.; Sato H.; Eberhardt P.; Nakayama Y.; Shiono T. Synthesis of Stereodiblock Polyisoprene Consisting of *Cis*-1,4 and *Trans*-1,4 Sequences by Using a Neodymium Catalyst: Change of the Stereospecificity Triggered by an Aluminum Compound. Polym. Chem. 2016, 7, 1239–1243. 10.1039/C5PY01872B.

[ref45] Phuphuak Y.; Bonnet F.; Stoclet G.; Bria M.; Zinck P. Isoprene Chain Shuttling Polymerisation between *Cis* and *Trans* Regulating Catalysts: Straightforward Access to a New Material. Chem. Commun. 2017, 53, 5330–5333. 10.1039/C7CC01016H. 28447679

[ref46] Arriola D. J.; Carnahan E. M.; Hustad P. D.; Kuhlman R. L.; Wenzel T. T. Catalytic Production of Olefin Block Copolymers via Chain Shuttling Polymerization. Science 2006, 312, 714–719. 10.1126/science.1125268. 16675694

[ref47] Sinsky M. S.; Bass R. G.; Connell J. W.; Hergenrother P. M. Poly(enamine-ketones) from aromatic diacetylenic diketones and aromatic diamines. J. Polym. Sci., Part A: Polym. Chem. 1986, 24, 2279–2295. 10.1002/pola.1986.080240922.

[ref48] Bass R. G.; Cooper E.; Hergenrother P. M.; Connell J. W. Poly(enonsulfides) from the addition of aromatic dithiols to aromatic dipropynones. J. Polym. Sci., Part A: Polym. Chem. 1987, 25, 2395–2407. 10.1002/pola.1987.080250907.

[ref49] Wilbur J. M. Jr; Bonner B. A. Synthesis of hydrogen-terminated aliphatic bis(ethynyl ketone)s and aliphatic poly(enamine-ketone)s and poly(enonesulfide)s. J. Polym. Sci., Part A: Polym. Chem. 1990, 28, 3747–3759. 10.1002/pola.1990.080281318.

[ref50] Jim C. K. W.; Qin A.; Lam J. W. Y.; Mahtab F.; Yu Y.; Tang B. Z. Metal-Free Alkyne Polyhydrothiolation: Synthesis of Functional Poly(vinylenesulfide)s with High Stereoregularity by Regioselective Thioclick Polymerization. Adv. Funct. Mater. 2010, 20, 1319–1328. 10.1002/adfm.200901943.

[ref51] Liu J.; Lam J. W. Y.; Jim C. K. W.; Ng J. C. Y.; Shi J.; Su H.; Yeung K. F.; Hong Y.; Faisal M.; Yu Y.; Wong K. S.; Tang B. Z. Thiol–Yne Click Polymerization: Regio- and Stereoselective Synthesis of Sulfur-Rich Acetylenic Polymers with Controllable Chain Conformations and Tunable Optical Properties. Macromolecules 2011, 44, 68–79. 10.1021/ma1023473.

[ref52] Wandel M. B.; Bell C. A.; Yu J.; Arno M. C.; Dreger N. Z.; Hsu Y.-H.; Pitto-Barry A.; Worch J. C.; Dove A. P.; Becker M. L. Concomitant control of mechanical properties and degradation in resorbable elastomer-like materials using stereochemistry and stoichiometry for soft tissue engineering. Nat. Commun. 2021, 12, 446 10.1038/s41467-020-20610-5. 33469013 PMC7815890

[ref53] Hsu Y.-H.; Dove A. P.; Becker M. L. Crosslinked Internal Alkyne-Based Stereo Elastomers: Polymers with Tunable Mechanical Properties. Macromolecules 2021, 54, 4649–4657. 10.1021/acs.macromol.1c00246.

[ref54] Hsu Y.-H.; Luong D.; Asheghali D.; Dove A. P.; Becker M. L. Shape Memory Behavior of Biocompatible Polyurethane Stereoelastomers Synthesized via Thiol–Yne Michael Addition. Biomacromolecules 2022, 23, 1205–1213. 10.1021/acs.biomac.1c01473. 35044744

[ref55] Cha C.; Kohman R. H.; Kong H. Biodegradable Polymer Crosslinker: Independent Control of Stiffness, Toughness, and Hydrogel Degradation Rate. Adv. Funct. Mater. 2009, 19, 3056–3062. 10.1002/adfm.200900865.

[ref56] Cha C.; Kim S. Y.; Cao L.; Kong H. Decoupled control of stiffness and permeability with a cell-encapsulating poly(ethylene glycol) dimethacrylate hydrogel. Biomaterials 2010, 31, 4864–4871. 10.1016/j.biomaterials.2010.02.059. 20347136

[ref57] Kharkar P. M.; Rehmann M. S.; Skeens K. M.; Maverakis E.; Kloxin A. M. Thiol-ene click hydrogels for therapeutic delivery. ACS Biomater. Sci. Eng. 2016, 2, 165–179. 10.1021/acsbiomaterials.5b00420. 28361125 PMC5369354

[ref58] Macdougall L. J.; Truong V. X.; Dove A. P. Efficient In Situ Nucleophilic Thiol-yne Click Chemistry for the Synthesis of Strong Hydrogel Materials with Tunable Properties. ACS Macro Lett. 2017, 6, 93–97. 10.1021/acsmacrolett.6b00857. 35632898

[ref59] Macdougall L. J.; Pérez-Madrigal M. M.; Arno M. C.; Dove A. P. Nonswelling Thiol–Yne Cross-Linked Hydrogel Materials as Cytocompatible Soft Tissue Scaffolds. Biomacromolecules 2018, 19, 1378–1388. 10.1021/acs.biomac.7b01204. 29125285 PMC5954353

[ref60] Truong V. X.; Tsang K. M.; Forsythe J. S. Nonswelling Click-Cross-Linked Gelatin and PEG Hydrogels with Tunable Properties Using Pluronic Linkers. Biomacromolecules 2017, 18, 757–766. 10.1021/acs.biomac.6b01601. 28195689

[ref61] Kamata H.; Kushiro K.; Takai M.; Chung U.-i.; Sakai T. Non-Osmotic Hydrogels: A Rational Strategy for Safely Degradable Hydrogels. Angew. Chem. Int. Ed. 2016, 55, 9282–9286. 10.1002/anie.201602610. 27320060

[ref62] Kamata H.; Akagi Y.; Kayasuga-Kariya Y.; Chung U.-I.; Sakai T. “Nonswellable” Hydrogel Without Mechanical Hysteresis. Science 2014, 343, 873–875. 10.1126/science.1247811. 24558157

[ref63] Macdougall L. J.; Wiley K. L.; Kloxin A. M.; Dove A. P. Design of synthetic extracellular matrices for probing breast cancer cell growth using robust cyctocompatible nucleophilic thiol-yne addition chemistry. Biomaterials 2018, 178, 435–447. 10.1016/j.biomaterials.2018.04.046. 29773227 PMC6699181

[ref64] Macdougall L. J.; Pérez-Madrigal M. M.; Shaw J. E.; Inam M.; Hoyland J. A.; O’Reilly R.; Richardson S. M.; Dove A. P. Self-healing, stretchable and robust interpenetrating network hydrogels. Biomater. Sci. 2018, 6, 2932–2937. 10.1039/C8BM00872H. 30238110

[ref65] Yu Y.; Wei Z.; Leng X.; Li Y. Facile preparation of stereochemistry-controllable biobased poly(butylene maleate-co-butylene fumarate) unsaturated copolyesters: a chemoselective polymer platform for versatile functionalization via aza-Michael addition. Polym. Chem. 2018, 9, 5426–5441. 10.1039/C8PY01051J.

[ref66] Chen T.; Tian S.; Xie Z.; Guo Z.-X.; Xu J.; Guo B.-H. Two new approaches based on dynamic carboxyl–hydroxyl or hydroxyl–carboxyl transformation for high molecular weight poly(butylene maleate). Polym. Chem. 2020, 11, 5884–5892. 10.1039/D0PY00863J.

[ref67] Tang T.; Moyori T.; Takasu A. Isomerization-Free Polycondensations of Cyclic Anhydrides with Diols and Preparation of Polyester Gels Containing *Cis* or *Trans* Carbon Double Bonds via Photo-Cross-Linking and Isomerization in the Gels. Macromolecules 2013, 46, 5464–5472. 10.1021/ma400875x.

[ref68] Kricheldorf H. R.; Yashiro T.; Weidner S. Isomerization-Free Polycondensations of Maleic Anhydride with α,ω-Alkanediols. Macromolecules 2009, 42, 6433–6439. 10.1021/ma9009356.

[ref69] Daglar O.; Ozcan B.; Gunay U. S.; Hizal G.; Tunca U.; Durmaz H. Extremely rapid postfunctionalization of maleate and fumarate main chain polyesters in the presence of TBD. Polymer 2019, 182, 121844 10.1016/j.polymer.2019.121844.

[ref70] DiCiccio A. M.; Coates G. W. Ring-Opening Copolymerization of Maleic Anhydride with Epoxides: A Chain-Growth Approach to Unsaturated Polyesters. J. Am. Chem. Soc. 2011, 133, 10724–10727. 10.1021/ja203520p. 21699247

[ref71] Stubbs C. J.; Worch J. C.; Prydderch H.; Becker M. L.; Dove A. P. Unsaturated Poly(ester-urethanes) with Stereochemically Dependent Thermomechanical Properties. Macromolecules 2020, 53, 174–181. 10.1021/acs.macromol.9b01700.

[ref72] Huang S.; Kim K.; Musgrave G. M.; Sharp M.; Sinha J.; Stansbury J. W.; Musgrave C. B.; Bowman C. N. Determining Michael acceptor reactivity from kinetic, mechanistic, and computational analysis for the base-catalyzed thiol-Michael reaction. Polym. Chem. 2021, 12, 3619–3628. 10.1039/D1PY00363A. 34484433 PMC8409055

[ref73] Nguyen L.-T. T.; Gokmen M. T.; Du Prez F. E. Kinetic comparison of 13 homogeneous thiol–X reactions. Polym. Chem. 2013, 4, 5527–5536. 10.1039/c3py00743j.

[ref74] Moon N. G.; Mazzini F.; Pekkanen A. M.; Wilts E. M.; Long T. E. Sugar-Derived Poly(β-thioester)s as a Biomedical Scaffold. Macromol. Chem. Phys. 2018, 219, 1800177 10.1002/macp.201800177.

[ref75] Chan J. W.; Hoyle C. E.; Lowe A. B.; Bowman M. Nucleophile-Initiated Thiol-Michael Reactions: Effect of Organocatalyst, Thiol, and Ene. Macromolecules 2010, 43, 6381–6388. 10.1021/ma101069c.

[ref76] Khalfa A. L.; Becker M. L.; Dove A. P. Stereochemistry-Controlled Mechanical Properties and Degradation in 3D-Printable Photosets. J. Am. Chem. Soc. 2021, 143, 17510–17516. 10.1021/jacs.1c06960. 34652902

[ref77] Stubbs C. J.; Dove A. P. Understanding structure–property relationships of main chain cyclopropane in linear polyesters. Polym. Chem. 2020, 11, 6251–6258. 10.1039/D0PY01004A.

[ref78] Saxon D. J.; Luke A. M.; Sajjad H.; Tolman W. B.; Reineke T. M. Next-generation polymers: Isosorbide as a renewable alternative. Prog. Polym. Sci. 2020, 101, 101196 10.1016/j.progpolymsci.2019.101196.

[ref79] Galbis J. A.; García-Martín M. d. G.; de Paz M. V.; Galbis E. Synthetic polymers from sugar-based monomers. Chem. Rev. 2016, 116, 1600–1636. 10.1021/acs.chemrev.5b00242. 26291239

[ref80] Fenouillot F.; Rousseau A.; Colomines G.; Saint-Loup R.; Pascault J. P. Polymers from renewable 1,4:3,6-dianhydrohexitols (isosorbide, isomannide and isoidide): A review. Prog. Polym. Sci. 2010, 35, 578–622. 10.1016/j.progpolymsci.2009.10.001.

[ref81] Park S.-A.; Jeon H.; Kim H.; Shin S.-H.; Choy S.; Hwang D. S.; Koo J. M.; Jegal J.; Hwang S. Y.; Park J.; Oh D. X. Sustainable and recyclable super engineering thermoplastic from biorenewable monomer. Nat. Commun. 2019, 10, 2601 10.1038/s41467-019-10582-6. 31197142 PMC6565616

[ref82] Gormong E. A.; Reineke T. M.; Hoye T. R. Synthesis of Isohexide Diyne Polymers and Hydrogenation to Their Saturated Polyethers. ACS Macro Lett. 2021, 10, 1068–1072. 10.1021/acsmacrolett.1c00422. 35549115 PMC12276944

[ref83] Lillie L. M.; Tolman W. B.; Reineke T. M. Degradable and renewably-sourced poly(ester-thioethers) by photo-initiated thiol–ene polymerization. Polym. Chem. 2018, 9, 3272–3278. 10.1039/C8PY00502H.

[ref84] Lillie L. M.; Tolman W. B.; Reineke T. M. Structure/property relationships in copolymers comprising renewable isosorbide, glucarodilactone, and 2,5-bis(hydroxymethyl)furan subunits. Polym. Chem. 2017, 8, 3746–3754. 10.1039/C7PY00575J.

[ref85] Shearouse W. C.; Lillie L. M.; Reineke T. M.; Tolman W. B. Sustainable Polyesters Derived from Glucose and Castor Oil: Building Block Structure Impacts Properties. ACS Macro Lett. 2015, 4, 284–288. 10.1021/acsmacrolett.5b00099. 35596338

[ref86] Petersen S. R.; Prydderch H.; Worch J. C.; Stubbs C. J.; Wang Z.; Yu J.; Arno M. C.; Dobrynin A. V.; Becker M. L.; Dove A. P. Ultra-Tough Elastomers from Stereochemistry-Directed Hydrogen Bonding in Isosorbide-Based Polymers. Angew. Chem. Int. Ed. 2022, 61, e202115904 10.1002/anie.202115904. PMC9311410 35167725

[ref87] Sheldon R. A. Metrics of green chemistry and sustainability: Past, present, and future. ACS Sustainable Chem. Eng. 2018, 6, 32–48. 10.1021/acssuschemeng.7b03505.

